# VPS13C regulates phospho-Rab10-mediated lysosomal function in human dopaminergic neurons

**DOI:** 10.1083/jcb.202304042

**Published:** 2024-02-15

**Authors:** Leonie F. Schrӧder, Wesley Peng, Ge Gao, Yvette C. Wong, Michael Schwake, Dimitri Krainc

**Affiliations:** 1 Department of Neurology, Northwestern University Feinberg School of Medicine, Chicago, IL, USA; 2 Biochemistry III/Faculty of Chemistry, Bielefeld University, Bielefeld, Germany

## Abstract

Loss-of-function mutations in *VPS13C* are linked to early-onset Parkinson’s disease (PD). While VPS13C has been previously studied in non-neuronal cells, the neuronal role of VPS13C in disease-relevant human dopaminergic neurons has not been elucidated. Using live-cell microscopy, we investigated the role of VPS13C in regulating lysosomal dynamics and function in human iPSC-derived dopaminergic neurons. Loss of VPS13C in dopaminergic neurons disrupts lysosomal morphology and dynamics with increased inter-lysosomal contacts, leading to impaired lysosomal motility and cellular distribution, as well as defective lysosomal hydrolytic activity and acidification. We identified Rab10 as a phospho-dependent interactor of VPS13C on lysosomes and observed a decreased phospho-Rab10-mediated lysosomal stress response upon loss of VPS13C. These findings highlight an important role of VPS13C in regulating lysosomal homeostasis in human dopaminergic neurons and suggest that disruptions in Rab10-mediated lysosomal stress response contribute to disease pathogenesis in VPS13C-linked PD.

## Teaser

Using live-cell microscopy, we find that loss of VPS13C in human neurons disrupts lysosomal morphology and dynamics with increased inter-lysosomal tethers, leading to impaired lysosomal motility and defective lysosomal function as well as a decreased phospho-Rab10-mediated lysosomal stress response.

## Introduction

Parkinson’s disease (PD) is one of the most common neurodegenerative disorders, which is characterized by the loss of dopaminergic neurons in the substantia nigra pars compacta (Poewe et al., 2017). Dysfunction in several cellular pathways involving lysosomes, mitochondria, and synapses have been implicated in PD pathogenesis, but the underlying disease mechanisms are still under investigation (Burbulla et al., 2017; Nguyen et al., 2019; Song et al., 2023). Rare compound heterozygous and homozygous mutations in vacuolar protein sorting 13 homolog C (*VPS13C*) were identified in early-onset PD patients (Darvish et al., 2018; Lesage et al., 2016; Smolders et al., 2021), who presented clinically with rapid disease progression, early cognitive decline (Lesage et al., 2016), severe neuronal loss in the substantia nigra, and diffuse Lewy body disease (Lesage et al., 2016; Smolders et al., 2021). However, as prior studies of VPS13C have been restricted to non-neuronal cells (Cai et al., 2022; Chen et al., 2022; Hancock-Cerutti et al., 2022; Hook et al., 2020; Kumar et al., 2018; Lesage et al., 2016; Yang et al., 2016), the underlying mechanisms of VPS13C-linked PD in human neurons have remained elusive.

Lysosomes are small, highly dynamic, and membrane-bound organelles with an acidic lumen that contains hydrolytic enzymes to facilitate the degradation of biological macromolecules. Other functions of lysosomes include acidification, intracellular transport, and membrane contact and fusion (Ballabio and Bonifacino, 2020; Roney et al., 2022). Lysosomal dysfunction is associated with several neurodegenerative diseases, emphasizing the importance of lysosomes in neuronal health (Roney et al., 2022). Recently, multiple studies have also suggested a role for phosphorylated Rab10 in lysosomal stress response pathways to maintain lysosomal homeostasis (Eguchi et al., 2018; Kuwahara et al., 2020). However, how VPS13C regulates neuronal lysosomal network dynamics and function and whether VPS13C interacts with Rab10 to regulate the lysosomal stress response have never been investigated, which has important implications for advancing our understanding of PD pathogenic mechanisms.

Here, we investigated the role of VPS13C on lysosomal dynamics and function in human-induced pluripotent stem cell (hiPSC)-derived dopaminergic neurons. Using high spatial and temporal resolution live-cell confocal microscopy, we observed markedly enlarged lysosomes in VPS13C-deficient human dopaminergic neurons. Enlarged lysosomes in VPS13C-deficient neurons formed increased inter-lysosomal contact tethers, leading to defective lysosomal motility and distribution. Importantly, we identified a preferential interaction between VPS13C and phosphorylated Rab10 through an unbiased screen for Rab10 phospho-dependent interactors. Interestingly, both high-resolution microscopy and lysosomal purification approaches further demonstrated that VPS13C’s interaction with Rab10 occurs on the lysosomal membrane, ultimately resulting in impaired phospho-Rab10-mediated lysosomal stress response upon loss of VPS13C. Lastly, VPS13C deficiency in human neurons significantly disrupted lysosomal function by impairing both lysosomal hydrolytic activity and acidification. Our work thus highlights a role of VPS13C in mediating lysosomal homeostasis in human dopaminergic neurons and suggests dysregulation of lysosomal dynamics and function as a key mechanism in VPS13C-linked PD.

## Results

### Loss of VPS13C disrupts lysosomal morphology in hiPSC-derived dopaminergic neurons

**Figure 1.**
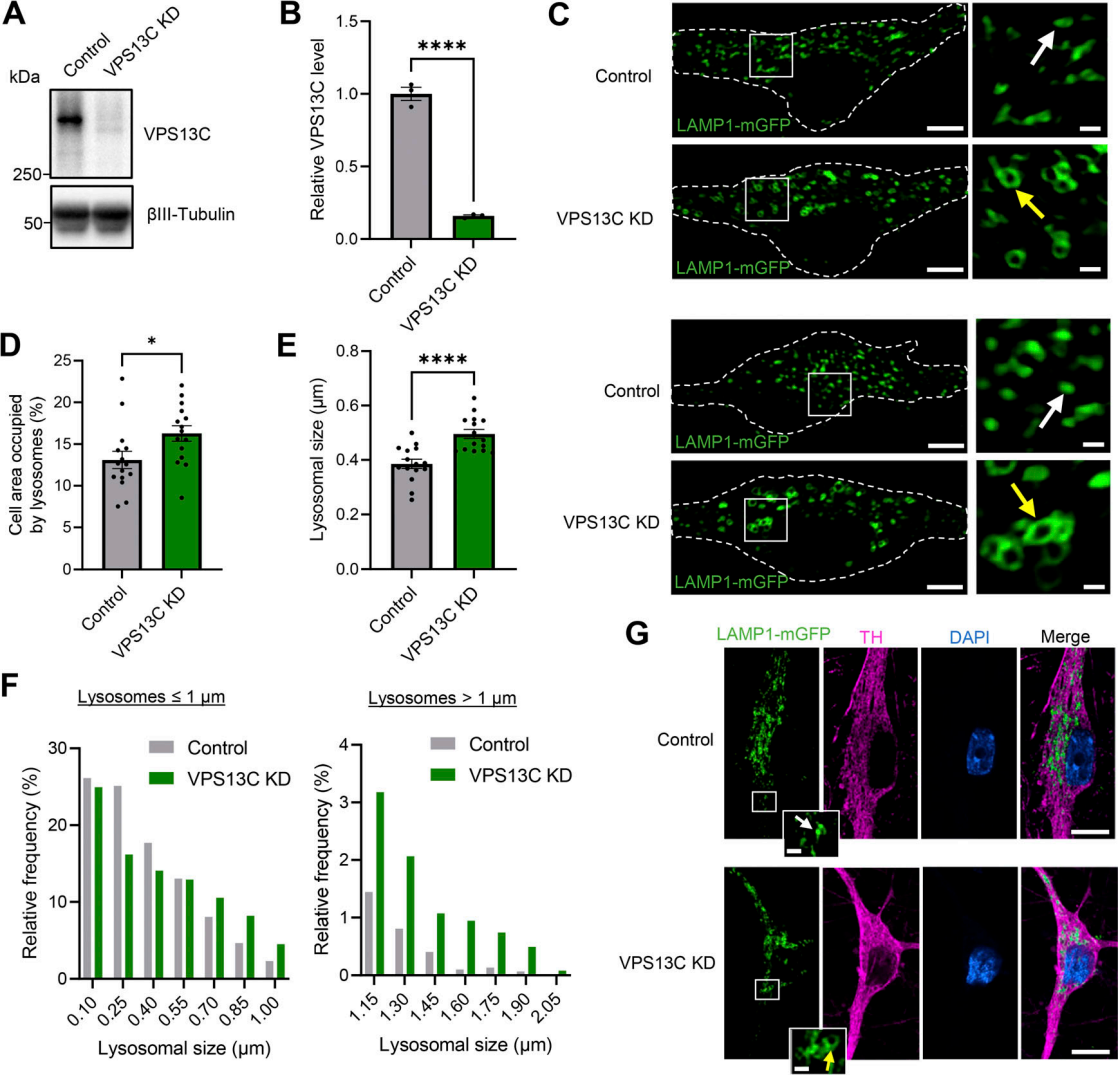
**Loss of VPS13C disrupts lysosomal morphology in hiPSC-derived dopaminergic neurons. (A and B)** Representative immunoblot and quantification of VPS13C KD efficiency in hiPSC-derived dopaminergic neurons (day 70) after 14 days of treatment with control and VPS13C shRNA. **(C)** Representative live-cell confocal images of LAMP1-mGFP (green) -positive vesicles in control (upper) and VPS13C KD (lower) neurons with insets showing smaller lysosomes (white arrow) in control condition versus enlarged lysosomes (yellow arrow) in VPS13C KD condition. Dashed line represents the outline of the cell (scale bar: 10 µm, inset: 1 µm). **(D)** Quantification of the percentage of cell area occupied by lysosomes from LAMP1-mGFP live-cell confocal imaging (*N* = 4, from *n* = 15 cells). **(E and F)** Quantification and histogram distribution of average lysosomal size in control and VPS13C KD dopaminergic neurons (*N* = 4, from *n* = 15 cells). **(G)** Representative images of PFA-fixed dopaminergic neurons with LAMP1-mGFP expression (green) and co-staining with TH (magenta) and DAPI (blue), with insets showing smaller lysosomes in control condition (upper panel, white arrow) and enlarged clustered lysosomes in VPS13C KD neurons (lower panel, yellow arrow) (scale bar: 10 µm, inset: 1 µm). Data are represented as mean ± SEM; unpaired two-tailed *t* test (B, D, and E); *P < 0.05 (D), ****P < 0.0001 (B and E). Source data are available for this figure: SourceData F1.

**Figure S1.**
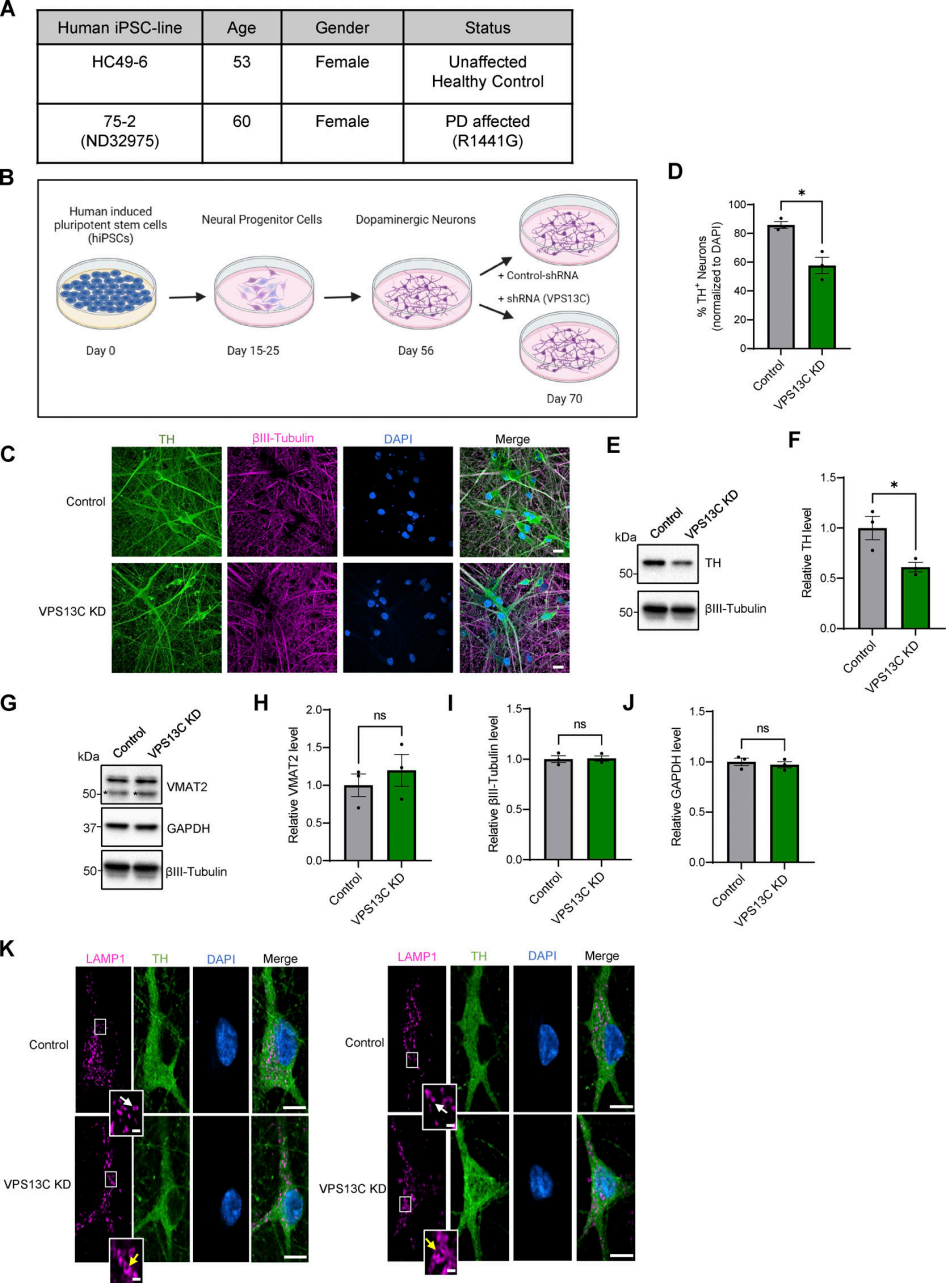
**Characterization of hiPSC-derived dopaminergic neurons in control and VPS13C KD condition. (A)** Table with information on the hiPSC lines obtained from Northwestern University Biorepository and Coriell Institute. **(B)** Schematic of the differentiation process from hiPSCs to dopaminergic neurons and treatment with lentiviral shRNA (14 days) for a non-targeting control and shRNA specifically targeting VPS13C at day 56 with MOI 2. Neurons were harvested on day 70 for the assessment of VPS13C KD efficiency and downstream readouts. Schematic was generated with http://BioRender.com. **(C)** Representative confocal images from fixed iPSC-derived dopaminergic control and VPS13C KD neurons showing immunostaining of TH (green), βIII-tubulin (magenta), and DAPI (blue) (scale bar: 20 µm). **(D)** Quantification of the percentage of TH-positive neurons (*N* = 3). **(E and F)** (E) Representative immunoblot of TH protein levels in control and VPS13C KD neurons and (F) relative quantification of TH levels. **(G–J)** Representative immunoblots and relative quantifications of neuronal synaptic marker VMAT2 (* unspecific protein band), neuronal marker βIII-tubulin, and GAPDH with relative quantifications (*N* = 3). **(K)** Representative confocal images of immunostaining for endogenous LAMP1 (magenta), TH (green), and DAPI (blue) in control neurons showing smaller LAMP1-positive vesicles (white arrow) in comparison to VPS13C KD neurons showing enlarged and clustered LAMP1-positive vesicles (yellow arrow) (D70) (scale bar: 10 µm, inset: 1 µm). Data represented as mean ± SEM; unpaired two-tailed *t* test (D, F, H, I, and J); ns: not significant (H, I, and J), *P < 0.05 (D and F). Source data are available for this figure: SourceData FS1.

VPS13C has previously been shown to localize to lysosomes (Cai et al., 2022; Kumar et al., 2018), but how VPS13C regulates lysosomal dynamics and function remains unclear. Notably, lysosomal dysfunction has been widely linked to PD (Navarro-Romero et al., 2020; Wallings et al., 2019) and thus could serve as a potential priming event in VPS13C-linked PD pathophysiology. To study the role of VPS13C in the endolysosomal system, we generated a VPS13C knockdown (KD) model in hiPSC-derived dopaminergic neurons (Fig. 1, A and B; and Fig. S1, A–J). Using live-cell confocal microscopy, we observed that LAMP1-positive vesicles, herein referred to as lysosomes, were significantly enlarged in VPS13C-deficient dopaminergic neurons (Fig. 1, C–E) with a substantial proportion of lysosomes being larger than 1 µm in diameter (Fig. 1 F). We further showed using immunofluorescence of the lysosomal marker (LAMP1-mGFP) (Fig. 1 G) and of endogenous LAMP1 (Fig. S1 K) that lysosomes were enlarged and more tethered in VPS13C-deficient tyrosine hydroxylase (TH)-positive dopaminergic neurons compared with control neurons, suggesting that loss of VPS13C disrupts lysosomal morphology in hiPSC-derived dopaminergic neurons.

### VPS13C deficiency disrupts inter-lysosomal contacts in hiPSC-derived dopaminergic neurons

**Figure 2.**
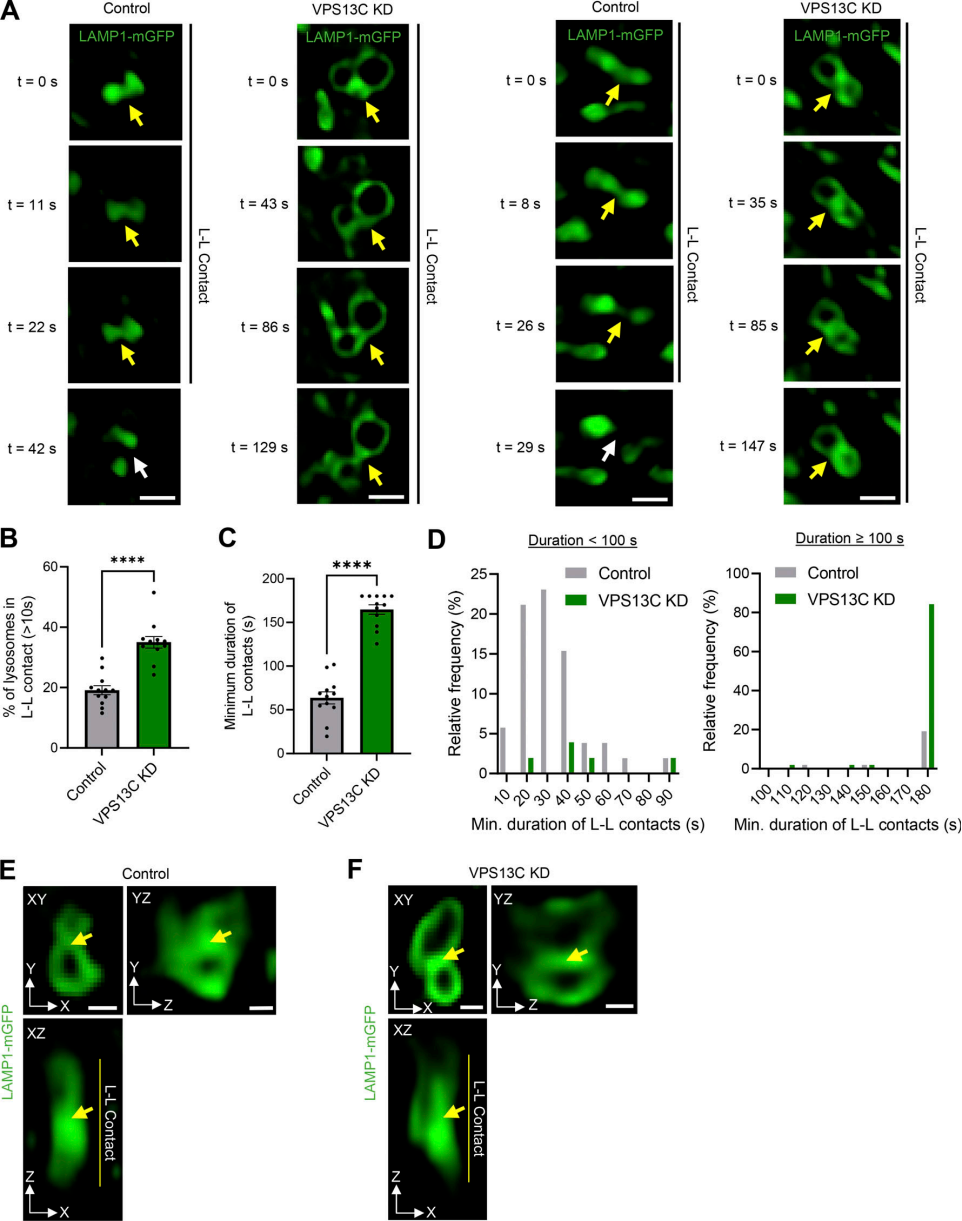
**VPS13C deficiency disrupts inter-lysosomal contacts in hiPSC-derived dopaminergic neurons. (A)** Time-lapse images of L-L contacts showing tethered lysosomes (yellow arrows) that untether at 42 and 29 s, respectively (white arrows), in control conditions but remain tethered in VPS13C KD neurons until 129 and 147 s, respectively. The majority of lysosomes in VPS13C KD neurons stay in contact until 180 s. Scale bar: 1 µm. **(B)** Quantification of the percentage of lysosomes in stable L-L contacts (≥10 s). **(C and D)** Quantification and histogram distribution of the minimum duration of L-L contacts. (*N* = 4, from *n* = 12 cells). **(E and F)** Representative 3D confocal images of L-L contacts in control neurons (E) and VPS13C KD neurons (F). Yellow arrows indicate the point of contact between two lysosomes in xy, yz, and xz projection (scale bar: 0.5 µm). Data represented as mean ± SEM; unpaired two-tailed *t* test (B and C); ****P < 0.0001 (B and C).

We further examined the effect of loss of VPS13C on lysosome-lysosome (L-L) contact site dynamics because the enlarged lysosomes in VPS13C-deficient neurons appeared to be more tethered than in control neurons (Fig. 2 A). VPS13C-deficient neurons had significantly more stable L-L contacts (Fig. 2 B) and prolonged L-L contact duration (Fig. 2, C and D). Indeed, the majority of L-L contacts in VPS13C-deficient neurons remained tethered for at least 180 s (Fig. 2, C and D). Additionally, we conducted 3D confocal live-cell imaging of inter-lysosomal contacts in both control neurons (Fig. 2 E) and in VPS13C KD neurons (Fig. 2 F), which further demonstrates that these inter-lysosomal contacts indeed form between lysosomal membranes.

**Figure S2.**
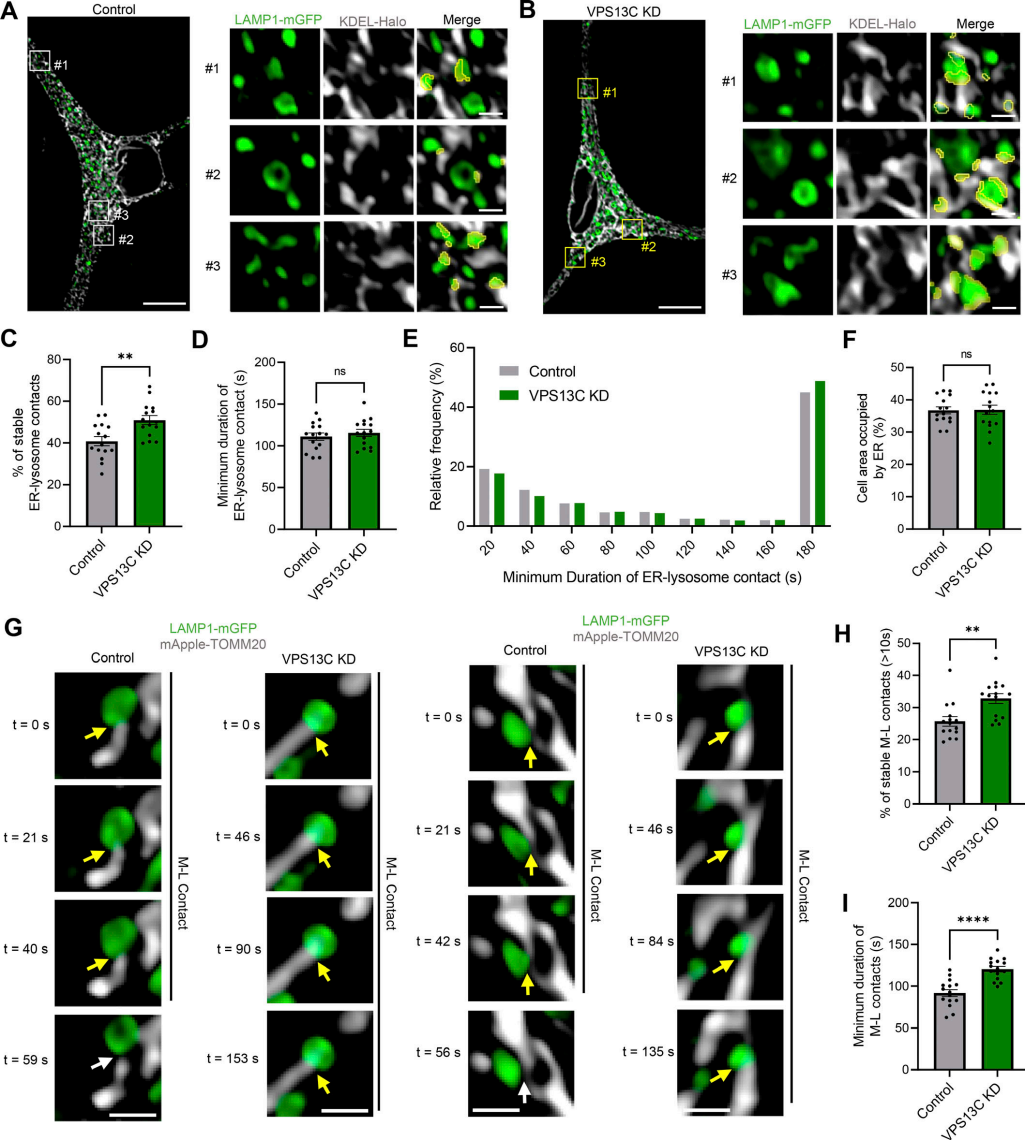
**VPS13C deficiency alters ER-lysosome and mitochondria-lysosome contact sites in iPSC-derived dopaminergic neurons. (A and B)** Representative live-cell confocal images showing lysosome-ER contact sites in control (A) and VPS13C KD (B) dopaminergic neurons expressing LAMP1-mGFP (green) and Halo-KDEL (gray) (scale bar: 10 µm, inset: 1 µm). **(C)** Quantification of the percentage of stable ER-lysosome contacts (≥10 s) (*N* = 4, from *n* = 15 cells). **(D and E)** Quantification and histogram distribution of the minimum duration of ER-lysosome contacts (*N* = 4, from *n* = 15 cells). **(F)** Quantification of the percentage of cell area occupied by ER (*N* = 4, from *n* = 15 cells). **(G)** Representative time-lapse confocal images showing mitochondria-lysosome contact sites in iPSC-derived dopaminergic neurons expressing LAMP1-mGFP (green) and mAppleTOMM20 (gray). Tethered mitochondria with lysosomes (yellow arrow) untether at 59 and 56 s, respectively, in control conditions (white arrow) but remain tethered in VPS13C KD neurons (yellow arrow) until 153 and 135 s, respectively (scale bar: 1 µm). **(H)** Quantification of the percentage of stable mitochondria-lysosome contacts (≥10 s) (*N* = 4, from *n* = 15 cells). **(I)** Quantification of the minimum contact duration of mitochondria-lysosome (M-L) contacts (*N* = 4, from *n* = 15 cells). Data represented as mean ± SEM; unpaired two-tailed *t* test (C, D, F, H, and I); ns: not significant (D and F), **P < 0.01 (C and H), ****P < 0.0001 (I).

To determine whether other inter-organelle contacts involving lysosomes were disrupted by loss of VPS13C, we examined ER-lysosome contacts given previous reports of VPS13C localization at these contact sites (Cai et al., 2022; Kumar et al., 2018). Using live-cell microscopy, we observed a significant increase in stable ER-lysosome contacts (Fig. S2, A–C), though the duration of ER-lysosome contacts was unchanged (Fig. S2, D and E). We did not observe any changes in ER morphology itself (Fig. S2, A, B, and F).

Recent studies have investigated mitochondria-lysosome contact sites that can regulate both mitochondrial and lysosomal dynamics (Wong et al., 2018, 2022) and are disrupted in iPSC-derived dopaminergic neurons with PD-linked mutations (Kim et al., 2021; Peng et al., 2023). Additionally, it was previously demonstrated that VPS13C KD in non-neuronal cells leads to mitochondrial morphology abnormalities (Lesage et al., 2016). Therefore, we examined the role of VPS13C in regulating mitochondria-lysosome contact site formation and tethering in additional live-cell microscopy studies (Fig. S2, G–I). We found that mitochondria-lysosome contacts were able to dynamically form in both control and VPS13C KD neurons (Fig. S2 G). However, the loss of VPS13C significantly increased the percentage of lysosomes forming contacts with mitochondria (Fig. S2 H) and significantly increased the tethering duration of mitochondria-lysosome contacts in VPS13C KD neurons (Fig. S2 I). Together, these findings demonstrate that L-L contacts as well as ER-lysosome and mitochondria-lysosome contact site dynamics are affected by loss of VPS13C.

### Increased lysosomal tethering affects lysosomal motility and distribution in VPS13C-deficient dopaminergic neurons

**Figure 3.**
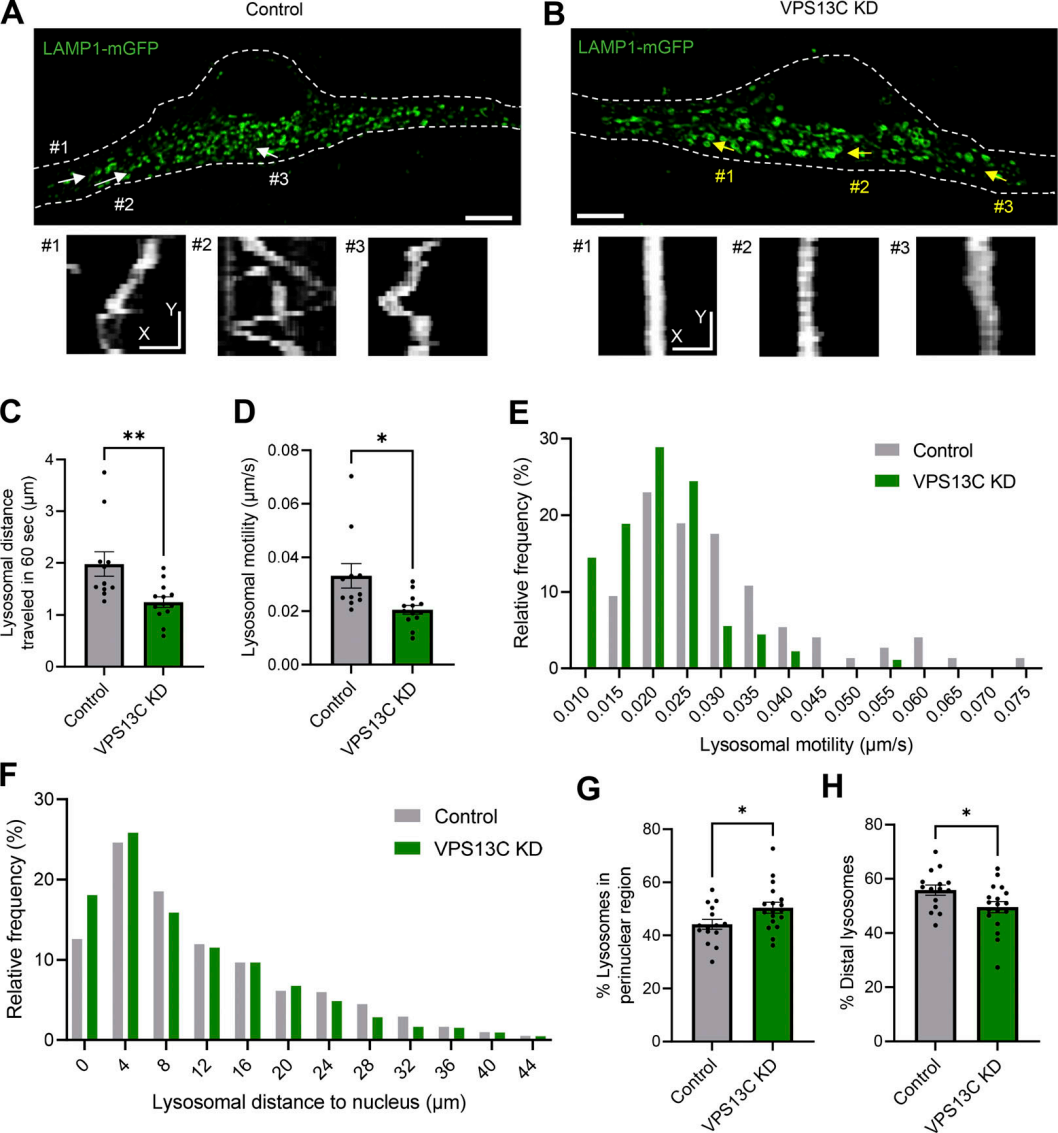
**Increased lysosomal tethering affects lysosomal motility and distribution in VPS13C-deficient dopaminergic neurons. (A and B)** Representative live-cell confocal images from control (A; Video 1) and VPS13C KD (B; Video 2) neurons showing LAMP1-positive vesicles (green, upper panel) and lysosomal motility demonstrated by kymographs (bottom panels). Dashed line represents the outline of the cell. Scale bar: 10 µm, kymograph scale bar: x = 1 µm, y = 20 s. **(C–E)** Quantification of the average distance traveled by lysosomes in 60 s and quantification of the average lysosomal motility with histogram distribution. *N* = 4, from *n* = 11 cells (control) and *n* = 13 cells (VPS13C KD). **(F)** Histogram distribution of the average lysosomal distance to the nucleus. *N* = 4, from *n* = 15 cells (control) and *n* = 18 cells (VPS13C KD). **(G)** Quantification of the percentage of lysosomes in the perinuclear region (<7 µm distance from nuclear membrane). **(H)** Quantification of distal lysosomes (≥7 µm distance from nuclear membrane). Data represented as mean ± SEM; unpaired two-tailed *t* test (C, D, G, and H); *P < 0.05 (D, G, and H), **P < 0.01 (C).

Inter-lysosomal contacts were recently demonstrated to regulate lysosomal network dynamics (Wong et al., 2022). Therefore, we investigated whether increased inter-lysosomal tethering in VPS13C-deficient neurons altered the motility and distribution of lysosomes. Indeed, we observed that lysosomes in VPS13C-deficient neurons had decreased motility compared with those in control neurons (Fig. 3, A–E; and Videos 1 and 2). Lysosomes in VPS13C-deficient neurons also demonstrated aberrant distribution with a significant proportion of lysosomes localized to the perinuclear region (Fig. 3, F and G). Consistent with this finding, VPS13C-deficient neurons exhibited fewer distal lysosomes (Fig. 3, F and H). Together, our data suggests that increased inter-lysosomal contact sites lead to aberrant lysosomal motility and preferential localization of lysosomes to the perinuclear region.

**Video 1.** **Lysosomal motility in control iPSC-derived dopaminergic neurons.** Example time-lapse video of lysosomal motility via LAMP1-mGFP signal in control human dopaminergic neurons. Corresponding to Fig. 3 A. Frame rate: 25 frames/second. Scale bar: 10 µm.

**Video 2.** **Lysosomal motility in VPS13C-deficient iPSC-derived dopaminergic neurons.** Example time-lapse video of lysosomal motility via LAMP1-mGFP signal in VPS13C KD human dopaminergic neurons. Corresponding to Fig. 3 B. Frame rate: 25 frames/second. Scale bar: 10 µm.

### VPS13C interacts with Rab10 in a phospho-dependent manner on the lysosomal membrane

To further investigate how loss of VPS13C causes lysosomal enlargement, we sought to identify potential interaction partners of VPS13C. Previous studies have suggested that VPS13C can interact with several Rab (Ras-associated binding) proteins (Gillingham et al., 2019; Hook et al., 2020; Huttlin et al., 2017), which are small GTPases that modulate intracellular membrane trafficking by cycling between the cytosol and different target membranes in response to changes in phosphorylation (Homma et al., 2021; Zerial and McBride, 2001). Given our data demonstrating defective lysosomal morphology and distribution upon loss of VPS13C, we were particularly interested in a possible interaction between VPS13C and Rab10, which has been shown to regulate lysosomal homeostasis (Eguchi et al., 2018) and lysosomal positioning (Kluss et al., 2022).

**Figure 4.**
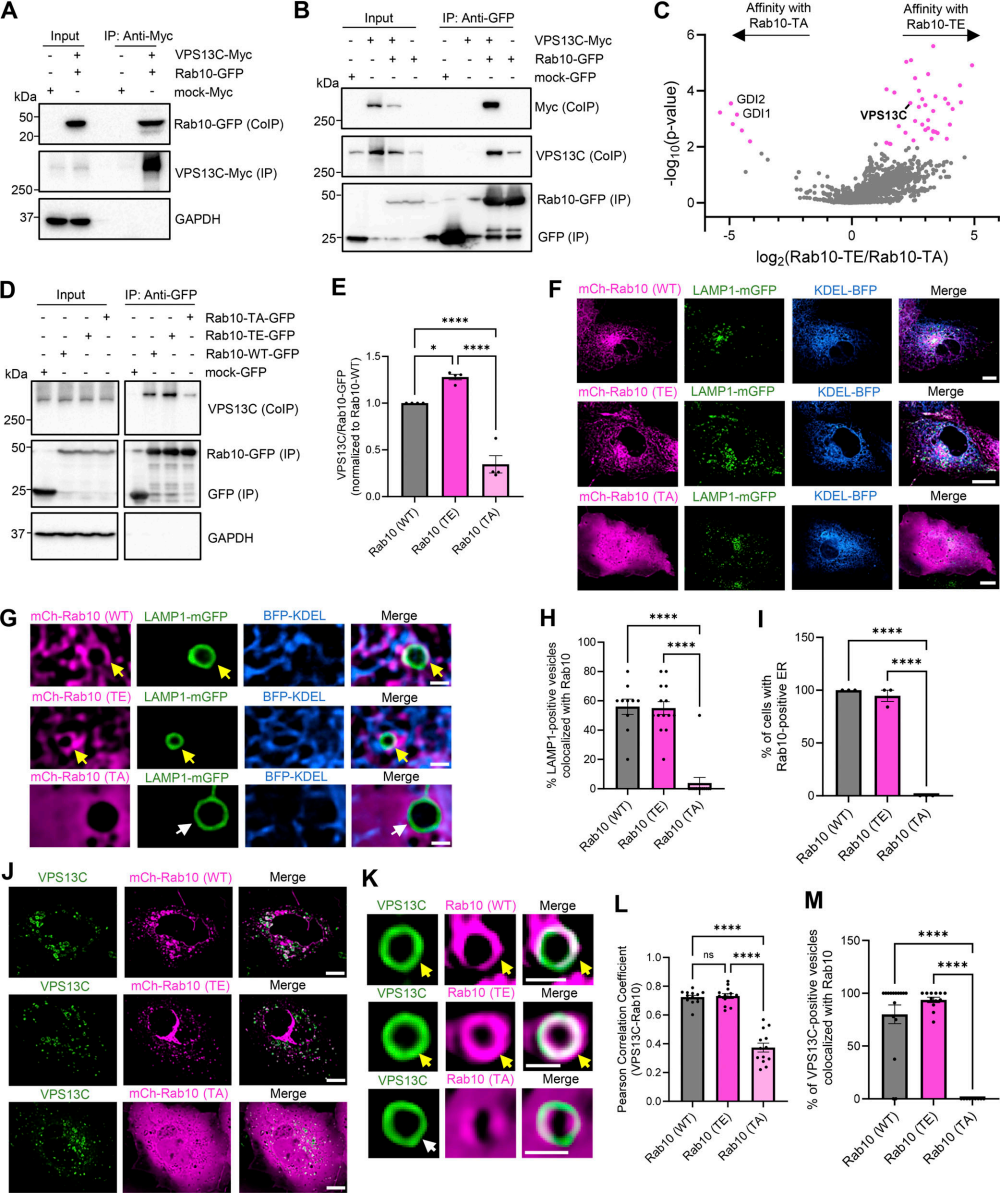
**VPS13C interacts with Rab10 in a phospho-dependent manner on the lysosomal membrane. (A)** Representative immunoblot of immunoprecipitated VPS13C-Myc and co-immunoprecipitated Rab10-GFP (*N* = 4). **(B)** Representative immunoblot of immunoprecipitated Rab10-GFP and co-immunoprecipitated VPS13C-Myc and endogenous VPS13C (*N* = 1). **(C)** Volcano plot of AP-MS data comparing interactors of phosphomimetic Rab10-T73E versus phosphodeficient Rab10-T73A (significant hits shown in magenta), highlighting previously confirmed interaction partners GDI1/2 show higher affinity for phosphodeficient Rab10-T73A, and newly identified interactor VPS13C show higher affinity for phosphomimetic Rab10-T73E (*N* = 3) (Data S1). **(D and E)** Confirmation of phospho-dependent interaction of Rab10-GFP and endogenous VPS13C via co-IP. Representative immunoblot of immunoprecipitated Rab10-GFP variants (WT, T73E, T73A) and co-immunoprecipitated endogenous VPS13C with (E) quantification of the VPS13C co-IP efficiency normalized by Rab10-GFP levels in IP fraction (*N* = 4). **(F and G)** Representative live-cell confocal images showing mCherry-Rab10 WT and TE, but not mCherry-Rab10 TA (magenta) colocalized with lysosomes (LAMP1-mGFP, green) and ER (BFP-KDEL, blue) in COS7 cells with zoom-out (F) and zoom-in (G) (scale bar: zoom-out: 10 µm, zoom-in: 1 µm). Yellow arrows indicate co-localization. **(H)** Quantification of the percentage of LAMP1-positive vesicles that colocalize with Rab10 (*N* = 3, from *n* > 10 cells). **(I)** Quantification of the percentage of cells with Rab10-positive ER (*N* = 3; from WT: *n* = 18 cells, T73E: *n* = 43 cells, T73A: *n* = 39 cells). **(J and K)** Representative live-cell confocal images showing colocalization of VPS13C-mClover (green) with Rab10 WT and Rab10 T73E, but not with Rab10 T73A (magenta) in COS7 cells (zoom-out [J]; zoom-in [K]) (scale bar: zoom-out: 10 µm, zoom-in: 1 µm). Yellow arrows indicate co-localization. **(L)** Pearson correlation coefficient of VPS13C and Rab10 colocalization comparing Rab10 WT, Rab10 T73E, and Rab10 T73A mutants (*N* = 3, from *n* = 13 cells). **(M)** Quantification of the percentage of VPS13C-positive vesicles colocalized with Rab10 WT, Rab10 T73E, and Rab10 T73A (*N* = 3, from *n* > 9 cells). Data are represented as mean ± SEM; one-way ANOVA with Tukey multiple comparison test (E, H, I, L, and M); *P < 0.05 (E), ****P < 0.0001 (E, H, I, L, and M). Source data are available for this figure: SourceData F4.

We thus investigated whether VPS13C interacts with Rab10 by conducting co-immunoprecipitation (co-IP) studies of Rab10 wild-type (WT) and phospho-variants, as Rab10 phosphorylation at position T73 has been further shown to modulate its regulation of lysosomes (Eguchi et al., 2018). In cells expressing Myc-tagged VPS13C and GFP-tagged Rab10, co-IP of Rab10 was observed with VPS13C (Fig. 4 A, lane 4). We confirmed this interaction by performing the reverse co-IP, which similarly showed co-IP of overexpressed VPS13C with Rab10 (Fig. 4 B, lane 7). Moreover, we observed co-IP of endogenous VPS13C with Rab10, further demonstrating an interaction between these proteins at physiological levels (Fig. 4 B, lane 8). To investigate if the interaction of VPS13C and Rab10 was modulated by Rab10’s phosphorylation state, GFP- and mCherry-tagged Rab10 phospho-variants were generated. We performed an unbiased affinity purification followed by mass spectrometry (AP-MS) screen using GFP-Rab10 WT, phosphomimetic GFP-Rab10 T73E, and phosphodeficient GFP-Rab10 T73A (Data S1). This approach was validated by identifying GDP-dissociation inhibitor 1 (GDI1) and GDI2, known Rab10 interactors, as significant hits with higher affinity for phosphodeficient Rab10 TA as expected (Chen et al., 2009; Steger et al., 2016). Interestingly, we discovered a preferential interaction of VPS13C with phosphomimetic Rab10 TE (Fig. 4 C). This phosphodependent interaction of VPS13C with phosphomimetic Rab10 TE was confirmed via co-IP (Fig. 4 D), which demonstrated increased co-IP of VPS13C with phosphomimetic Rab10 TE compared with phosphodeficient Rab10 TA (Fig. 4 E), further highlighting VPS13C’s preferential interaction with phosphorylated Rab10.

**Figure S3.**
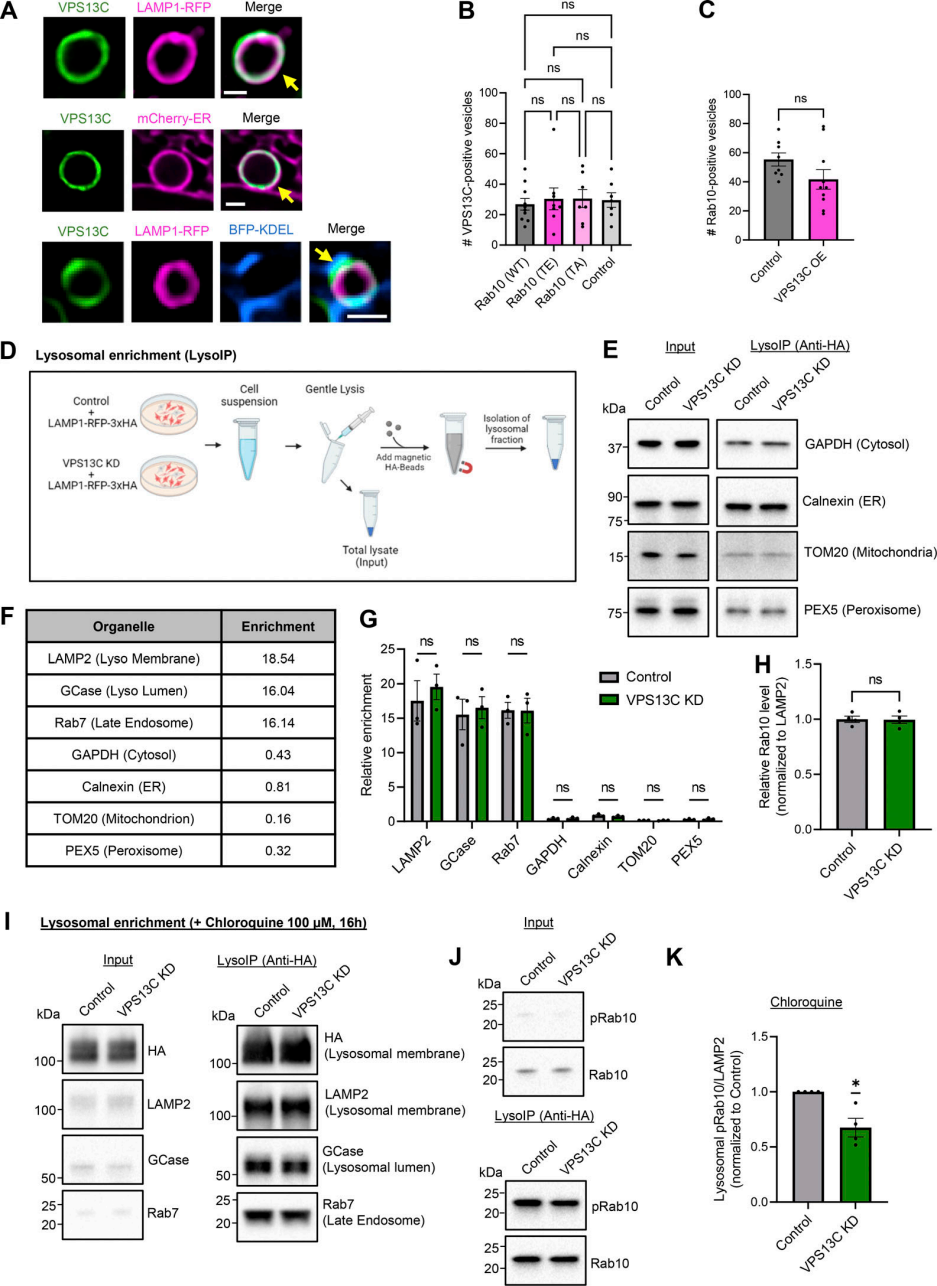
**Loss of VPS13C impairs phospho-Rab10-mediated lysosomal stress response. (A)** Representative live-cell confocal images showing localization of VPS13C (VPS13C^mClover3, green) on the lysosomal membrane (LAMP1-RFP, magenta) and at lysosome-ER contact sites (LAMP1-RFP and mCherry-ER [magenta] or BFP-KDEL [blue]). Yellow arrows indicate co-localization. *N* = 3, from *n* ≥ 20 cells. Scale bar: 1 µm. **(B)** Quantification of the number of VPS13C-positive vesicles with or without overexpression of Rab10 WT, Rab10 T73E, and Rab10 T73A (*N* = 3, from *n* > 10 cells). **(C)** Quantification of the number of Rab10-positive vesicles with and without overexpression of VPS13C (*N* = 3, from *n* > 10 cells). VPS13C and Rab10 do not regulate each other’s recruitment. **(D)** Schematic of the isolation and enrichment of lysosomes from HEK-293 FT cells expressing LAMP1-RFP-3xHA. Pulldown of lysosomes via anti-HA-magnetic beads. Schematic was generated with http://BioRender.com. **(E)** Representative immunoblot of protein markers for cytosol, ER, mitochondria, and peroxisomes in enriched lysosomal fractions. **(F and G)** Quantification of relative enrichment of lysosomal markers compared with other organelle markers from LysoIP fractions showing the purity in lysosomal fractions (*N* = 3). **(H)** Quantification of total Rab10 protein levels from lysosomal fractions (*N* = 4). **(I)** Representative immunoblot from LysoIP after CQ treatment (100 µM, 16 h) showing lysosomal enrichment. **(J)** Representative immunoblots of phospho-Rab10 (pRab10) and total Rab10 protein levels from LysoIP after CQ treatment. **(K)** Quantification of lysosomal phospho-Rab10 protein levels (*N* = 4). The effect of VPS13C KD on lysosomal phospho-Rab10 under CQ-induced lysosomal stress is comparable with that under basal conditions. Data represented as mean ± SEM; one-way ANOVA with Tukey multiple comparison test (B), unpaired two-tailed *t* test (C and H), multiple unpaired *t* test (G), one-sample *t* test (K); ns: not significant (B, C, G, and H), *P < 0.05 (K). Source data are available for this figure: SourceData FS3.

Next, we investigated the subcellular localization of Rab10 and VPS13C by first examining the role of phosphorylation on Rab10 localization. Using mCherry-tagged Rab10 phospho-variants, we found that both Rab10 WT and phosphomimetic Rab10 TE colocalized with late endosomal/lysosomal marker LAMP1-mGFP and ER marker BFP-KDEL. In contrast, phosphodeficient Rab10 TA was cytosolic with low levels of colocalization with LAMP1 and KDEL (Fig. 4, F–I). These findings suggested a potential shared intracellular localization of phosphorylated Rab10 and VPS13C, which also preferentially localized to lysosomes and ER (Fig. S3 A), as reported in recent studies (Cai et al., 2022; Kumar et al., 2018). Indeed, using live-cell confocal microscopy, we observed strong colocalization of VPS13C-mClover with both Rab10 WT and phosphomimetic Rab10 TE, but not with phosphodeficient Rab10 TA (Fig. 4, J–M). We also examined whether Rab10 WT or mutants altered the amount of VPS13C recruitment to vesicles. The number of VPS13C-positive vesicles was not altered by overexpression of Rab10 WT, phosphomimetic Rab10 TE, or phosphodeficient Rab10 TA (Fig. S3 B). Conversely, we also examined whether VPS13C altered the amount of Rab10 recruitment to vesicles. Similarly, the number of Rab10 WT-positive vesicles was not altered by overexpression of VPS13C (Fig. S3 C). Taken together, our results demonstrate that while VPS13C and Rab10 do not regulate each other’s recruitment, VPS13C preferentially colocalizes and interacts with phosphorylated Rab10 on lysosomes.

### Endogenous interaction between VPS13C and Rab10 depends on LRRK2-mediated Rab10 phosphorylation

**Figure 5.**
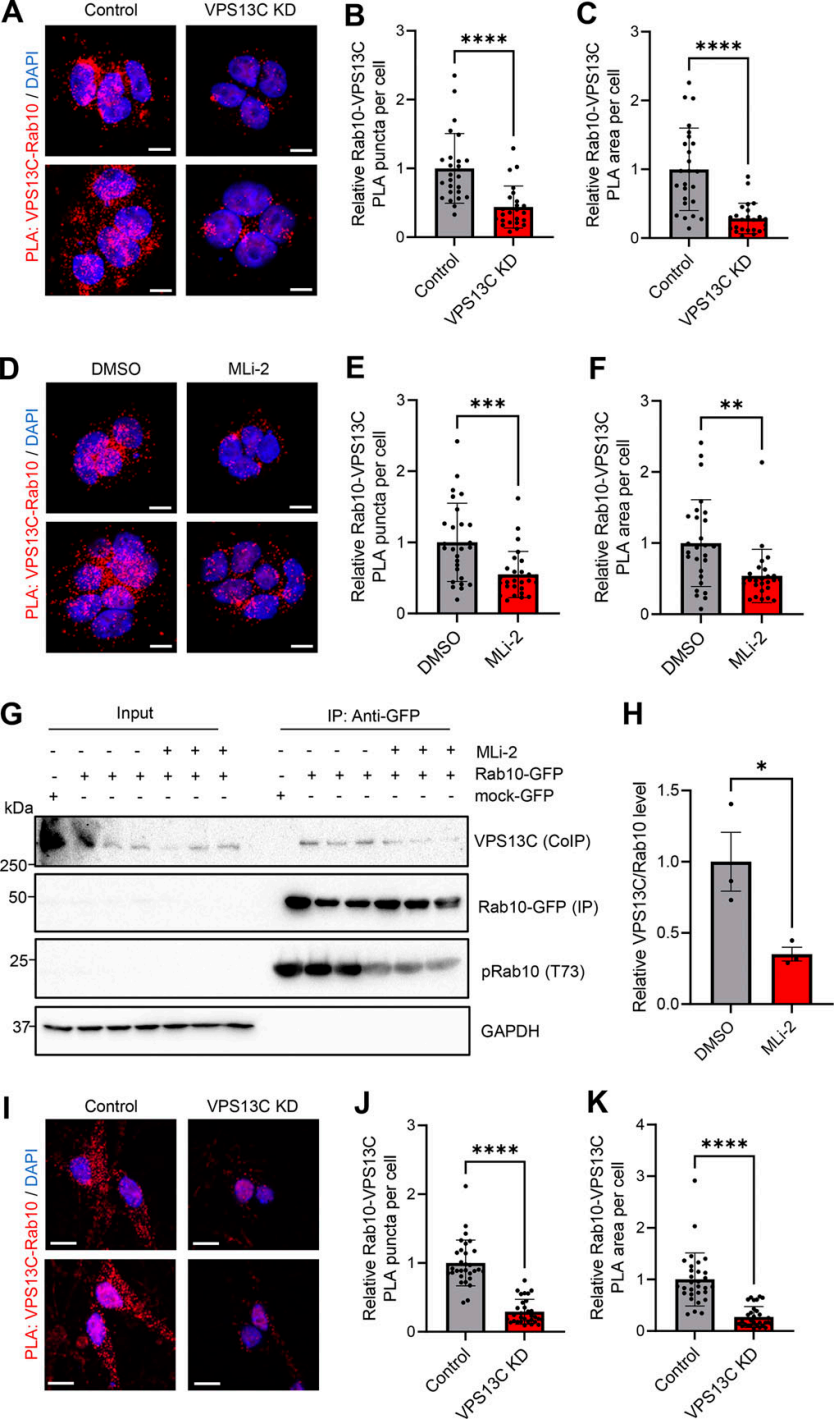
**Endogenous interaction between VPS13C and Rab10 depends on LRRK2-mediated Rab10 phosphorylation. (A)** Representative confocal images showing PLA signal (red) from VPS13C and Rab10 in control (left) and VPS13C KD (right) cells with DAPI staining (blue) (scale bar: 10 µm). **(B and C)** Relative quantification of PLA puncta per cell (B) and PLA area per cell (C) (*N* = 3, from *n* = 25 images [control] and *n* = 24 images [VPS13C KD]), confirming the interaction between VPS13C and Rab10 under physiological conditions. **(D)** Representative confocal images showing PLA signal (red) from MLi-2 treated control cells in comparison to vehicle (DMSO) treatment (scale bar: 10 µm). **(E and F)** Relative quantification of PLA punta per cell (E) and PLA area per cell (F) (*N* = 3, from *n* = 27 images), which validates the phospho-dependent interaction between VPS13C and Rab10 and indicates that the interaction depends on LRRK2-kinase activity. **(G)** Representative immunoblot of immunoprecipitated Rab10-GFP and co-immunoprecipitated endogenous VPS13C after treatment with MLi-2 in HEK293 FT cells. **(H)** Relative quantification of co-IP efficiency of VPS13C with or without MLi2 treatment (*N* = 3). **(I)** Representative confocal images showing PLA signal (red) from VPS13C and Rab10 in control (left) and VPS13C KD (right) dopaminergic neurons (scale bar: 10 µm). **(J and K)** Relative quantification of PLA puncta per cell (J) and PLA area per cell (K) (*N* = 4, from *n* = 30 images [control] and *n* = 31 images [VPS13C KD]), confirming that VPS13C and Rab10 interact in dopaminergic neurons. Data represented as mean ± SEM; unpaired two-tailed *t* test (B, C, E, F, H, J, and K); *P < 0.05 (H), **P < 0.01 (F), ***P < 0.001 (E), ****P < 0.0001 (B, C, J, and K). Source data are available for this figure: SourceData F5.

To further demonstrate the phospho-dependent interaction between VPS13C and Rab10 under physiological conditions, we used proximity ligation assays (PLA), which has previously been used to confirm interactions of endogenous proteins in close proximity (40 nm) to each other (Ito et al., 2018; Kuwahara et al., 2020; Quitterer et al., 2019). We demonstrated that the VPS13C-Rab10 PLA was specific to VPS13C and Rab10’s interaction and that it was significantly decreased in VPS13C KD cells (Fig. 5, A–C) Importantly, we further showed that inhibition of LRRK2 kinase activity by MLi-2 treatment led to significantly reduced PLA signal between VPS13C and Rab10 (Fig. 5, D–F), further supporting a phospho-dependent interaction and also suggesting that this interaction depends on LRRK2 kinase activity. Furthermore, we confirmed this by co-IP studies and found that inhibition of LRRK2 kinase activity by MLi-2 led to significantly reduced co-IP of VPS13C with Rab10-GFP (Fig. 5, G and H). Finally, we confirmed the endogenous interaction between VPS13C and Rab10 in iPSC-derived dopaminergic neurons (Fig. 5, I–K). Together, these findings demonstrate that VPS13C and Rab10 interact under physiological conditions and that LRRK2 kinase activity is an important regulator of this interaction.

### VPS13C deficiency decreases lysosomal phospho-Rab10 and impairs phospho-Rab10-mediated lysosomal stress response

**Figure 6.**
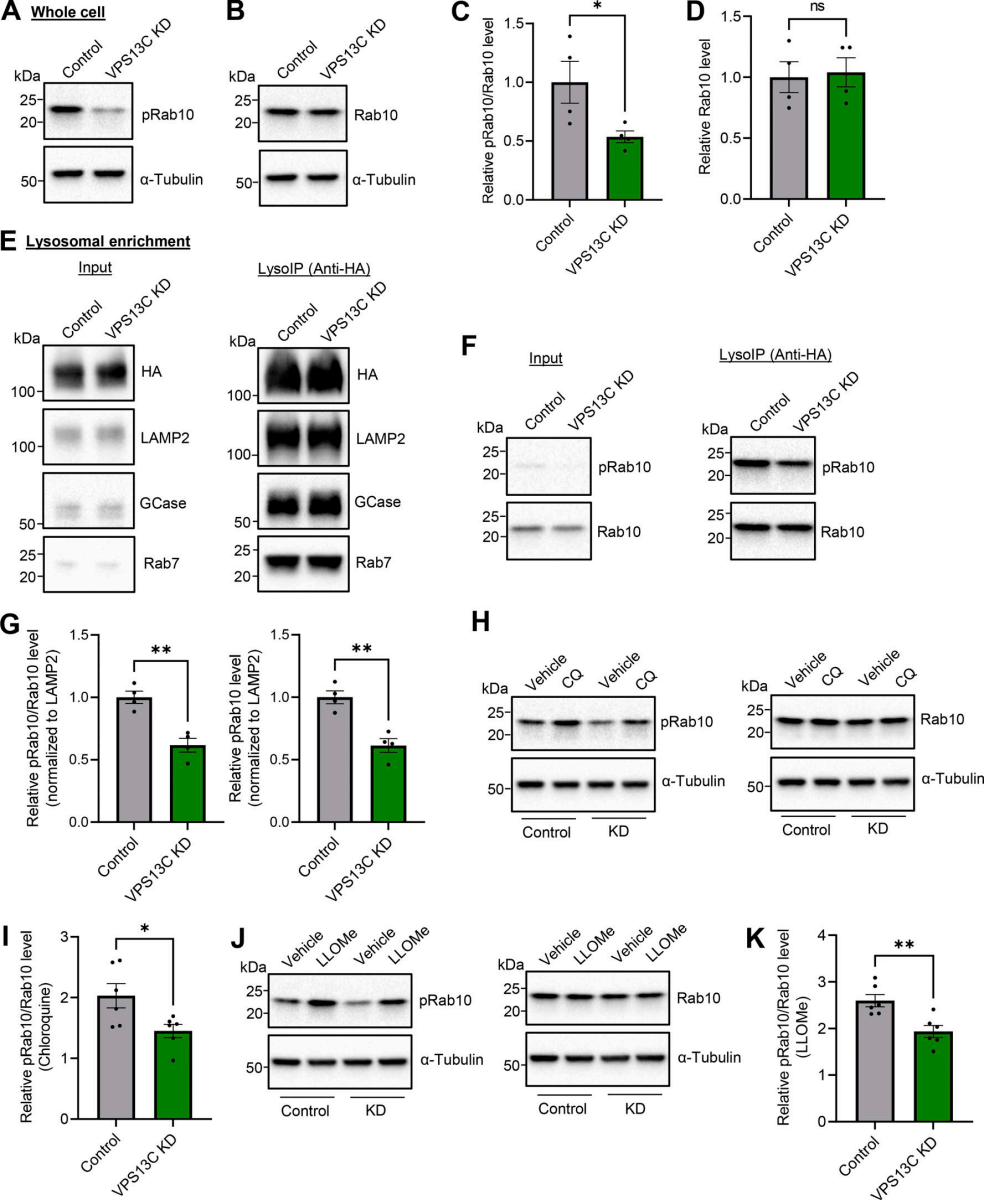
**VPS13C deficiency decreases lysosomal phospho-Rab10 and impairs phospho-Rab10-mediated lysosomal stress response. (A–D)** Representative immunoblot showing phospho-Rab10 (pRab10; T73) (A) and total Rab10 (B) protein levels from whole-cell lysates of HEK-293 FT cells and (C and D) relative quantification of pRab10 and total Rab10 protein levels (*N* = 4). **(E)** Representative immunoblot of lysosomal enrichment using LysoIP (anti-HA magnetic beads for LAMP1-RFP-3xHA pulldown). Immunoblot showing lysosomal enrichment efficiency using antibodies against HA, LAMP2 as a lysosomal membrane marker, and GCase as a luminal lysosome marker (equal loading of input and LysoIP fraction) (*N* = 4). **(F and G)** (F) Representative immunoblot of pRab10 and total Rab10 protein levels from LysoIP and (G) quantification of protein levels from LysoIP fractions (*N* = 4). **(H–K)** Representative immunoblot of pRab10 and total Rab10 protein levels in HEK-293 FT cells treated with the lysosomotropic compound CQ (H) (100 µM, 16 h) or pH-independent lysosomal stressor LLOMe (J) (500 µM, 1 h). Relative quantification of pRab10 normalized to total Rab10 protein levels under CQ (I) or LLOMe (K) treatment (*N* = 6). Data represented as mean ± SEM; unpaired two-tailed *t* test (C, D, G, I, and K); ns: not significant (D), *P < 0.05 (C and I), **P < 0.01 (G and K). Source data are available for this figure: SourceData F6.

Having shown a phospho-dependent interaction between VPS13C and Rab10, we next investigated whether loss of VPS13C directly affects Rab10 levels. Surprisingly, we found that phospho-Rab10 (T73) protein levels were significantly lower in VPS13C-deficient cells compared with control cells, while total Rab10 levels were unaffected (Fig. 6, A–D). Given our data suggesting preferential lysosomal localization of phospho-Rab10, we performed lysosomal immunoprecipitations (LysoIP) (Abu-Remaileh et al., 2017) to further interrogate lysosomal phospho-Rab10 levels in response to loss of VPS13C (Fig. S3 D). We successfully isolated lysosomes at high purity, achieving a near 20-fold enrichment of lysosomal membrane and luminal marker compared with other organelles (Fig. 6 E and Fig. S3, E–G). Consistent with findings from whole cell lysates, we found that lysosomal phospho-Rab10 levels were significantly reduced in VPS13C-deficient cells (Fig. 6, F and G), whereas total lysosomal Rab10 levels were unaffected (Fig. S3 H).

Recent studies have elucidated a pathway whereby phosphorylated Rab10 accumulates onto damaged lysosomes to mitigate lysosomal enlargement in response to lysosomal stress (Eguchi et al., 2018). Because VPS13C-deficient cells exhibited enlarged lysosomes with decreased lysosomal phospho-Rab10, we hypothesized that loss of VPS13C disrupts the phospho-Rab10-mediated response to lysosomal stress. To investigate how VPS13C-deficient cells respond to lysosomal stress, we treated control and VPS13C KD cells with the lysosomotropic compound chloroquine (CQ), which induces lysosomal stress via deacidification, leading to increased lysosomal phospho-Rab10 levels (Eguchi et al., 2018; Kuwahara et al., 2020). While control cells exhibited increased phospho-Rab10 levels after CQ treatment as expected, VPS13C-deficient cells demonstrated a significant reduction in phospho-Rab10 levels in comparison with control cells in response to CQ (Fig. 6, H and I), suggesting an impaired phospho-Rab10-mediated lysosomal stress response. Furthermore, we tested the effect of the pH-independent lysosomal stressor LLOMe on Rab10 phosphorylation, which showed a similar reduction in phospho-Rab10 levels in VPS13C KD conditions upon lysosomal stress treatment with LLOMe (Fig. 6, J and K) Accordingly, phospho-Rab10 levels were decreased in the lysosomal fractions from VPS13C-deficient cells treated with CQ (Fig. S3, I–K).

**Figure S4.**
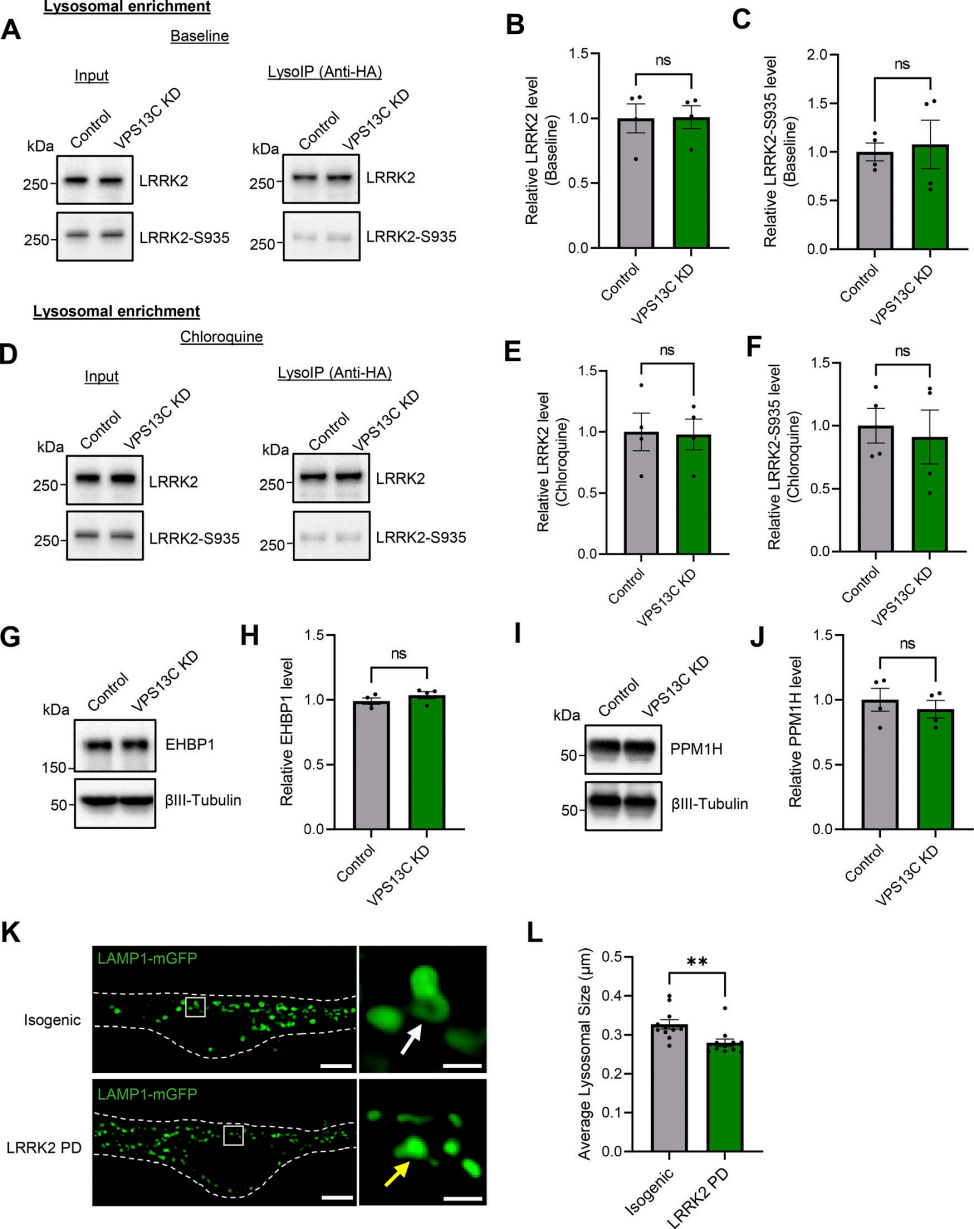
**VPS13C KD does not influence LRRK2 recruitment to lysosomes or its kinase activity. (A–C)** Representative immunoblots and relative quantification of LRRK2 and LRRK2-S935 protein levels in LysoIP fractions at baseline condition (*N* = 4). **(D–F)** Representative immunoblots and relative quantifications of LRRK2 and LRRK2-S935 protein levels in lysosomal fractions after treatment with CQ (100 μM, 16 h) (*N* = 4). LRRK2 and LRRK2-S935 protein levels in lysosomal fractions are unchanged at baseline and under lysosomal stress conditions. **(G and H)** Representative immunoblot and relative protein quantification of Rab10 downstream effector protein EHBP1. **(I and J)** Representative immunoblot and relative protein quantification of Rab10 phosphatase PPM1H. **(K)** Representative live-cell confocal images of LAMP1-mGFP (green) in LRRK2 PD mutant (R1441G, lower) and isogenic control (upper) dopaminergic neurons with insets showing smaller lysosomes (yellow arrow) in LRRK2 PD-mutant neurons in comparison to the isogenic control (white arrow) (scale bar: 10 µm, inset: 1 µm). Dashed line represents the outline of the cell. **(L)** Quantification of average lysosomal size in isogenic control and LRRK2 PD-mutant neurons (*N* = 3, from *n* = 11 cells). Data represented as mean ± SEM; unpaired two-tailed *t* test (B, C, E, F, H, J, and L); ns: not significant (B, C, E, F, H, and J), **P < 0.01 (L). Source data are available for this figure: SourceData FS4.

Additionally, we tested whether VPS13C KD affects the recruitment of LRRK2 to lysosomes and its activity. We first examined whether LRRK2 recruitment to lysosomes was altered by loss of VPS13C by examining LRRK2 levels in purified lysosomes in control and VPS13C KD conditions. Specifically, LRRK2 levels in LysoIP fractions were not altered in VPS13C KD cells (Fig. S4, A and B), demonstrating that VPS13C does not regulate LRRK2 recruitment to lysosomes at baseline conditions. Next, we examined whether LRRK2 activity was altered by loss of VPS13C by examining LRRK2 phosphorylation in control and VPS13C KD conditions. Of note, LRRK2-Ser935 phosphorylation levels in LysoIP fractions were not altered in VPS13C KD cells (Fig. S4, A and C), thus demonstrating that VPS13C does not regulate LRRK2 recruitment or activity. Finally, we also increased LRRK2 recruitment to lysosomes using CQ treatment. Under these conditions, we examined whether LRRK2 levels and activity in lysosomes were altered upon VPS13C KD to determine if these changes might regulate Rab10 phosphorylation. We found that LRRK2 levels in LysoIP fractions were not altered between control and VPS13C KD cells upon CQ treatment (Fig. S4, D and E) and that LRRK2-Ser935 phosphorylation levels in LysoIP fractions were also not changed between control and VPS13C KD cells upon CQ treatment (Fig. S4, D and F). Together, these results demonstrate that VPS13C does not affect the recruitment of LRRK2 or its kinase activity on lysosomes, and further demonstrate that reduced phospho-Rab10 levels in VPS13C KD cells are not due to changes in LRRK2’s recruitment to lysosomes or its activity.

We also examined whether VPS13C deficiency affects the protein levels of Rab10’s downstream effector protein EHBP1, which is involved in lysosomal homeostasis (Eguchi et al., 2018), and of PPM1H, which is a known phosphatase of Rab10 (Berndsen et al., 2019). We did not observe any changes in levels of either EHBP1 or PPM1H in VPS13C-deficient cells in comparison with control cells (Fig. S4, G–J), suggesting that lower phospho-Rab10 levels in VPS13C KD conditions are not due to increased dephosphorylation of Rab10 but rather a direct effect of VPS13C.

Finally, we investigated the effect of increased Rab10 phosphorylation via increased LRRK2 activity in LRRK2 PD patient neurons on lysosomal size. Specifically, we conducted live-cell confocal imaging studies in iPSC-derived dopaminergic neurons from LRRK2 PD mutant (R1441G) in comparison with isogenic control neurons (Fig. S4 K). Importantly, lysosomes were significantly smaller in LRRK2 PD mutant neurons (Fig. S4 L), suggesting that levels of phosphorylated Rab10 regulate lysosomal size in iPSC-derived dopaminergic neurons.

Altogether, these findings suggest that VPS13C is critical for regulating phospho-Rab10 protein levels on lysosomes and for the phospho-Rab10-mediated lysosomal stress response.

### Loss of VPS13C impairs lysosomal hydrolytic activity and acidification in dopaminergic neurons

**Figure 7.**
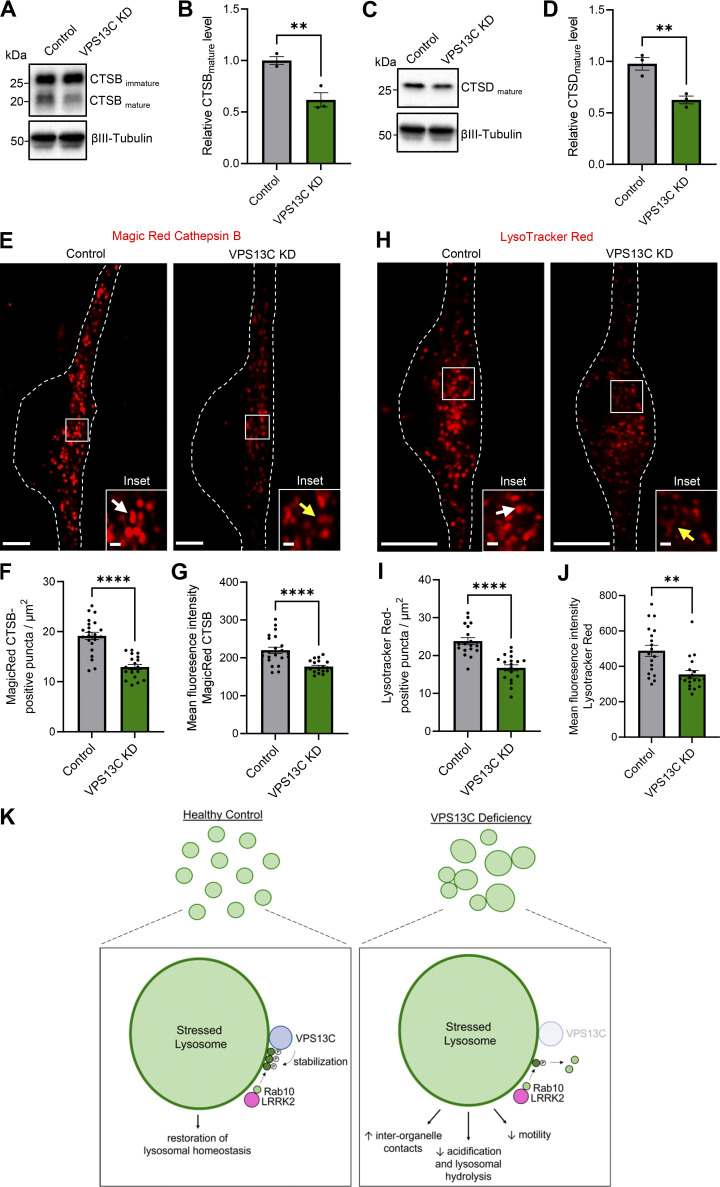
**Loss of VPS13C impairs lysosomal hydrolytic activity and acidification in dopaminergic neurons. (A–D)** Representative immunoblots showing protein levels of mature cathepsin B (CTSB) (A) and mature cathepsin D (CTSD) (C) with relative protein quantifications, respectively (B and D) (*N* = 3). **(E)** Representative live-cell confocal images of Magic Red cathepsin B staining in control neurons showing stronger cathepsin B intensity (white arrow) and in VPS13C KD neurons showing lower cathepsin B intensity (yellow arrow). Scale bar: 10 µm, inset: 1 µm. Dashed line represents the outline of the cell. **(F and G)** Quantification of the number of Magic Red cathepsin B-positive puncta per cell area (F) and the mean fluorescence intensity of Magic Red cathepsin B-positive puncta (G) (*N* = 4, from *n* = 22 cells [control] and *n* = 19 cells [VPS13C KD]). **(H)** Representative live-cell confocal images of LysoTracker Red DND-99 staining in control neurons showing stronger LysoTracker intensity (white arrow) and in VPS13C KD neurons showing reduced LysoTracker intensity (yellow arrow). Scale bar: 10 µm, inset: 1 µm. Dashed line represents the outline of the cell. **(I and J)** Quantification of the number of LysoTracker Red–positive puncta per cell area (I) and the mean fluorescence intensity of LysoTracker Red-positive puncta (J) (*N* = 4, from *n* = 19 cells). **(K)** Schematic of lysosomal phenotypes in VPS13C deficiency in comparison with healthy control. VPS13C-deficient cells have enlarged lysosomes that tether together, are less motile, and have impaired lysosomal hydrolytic activity and acidification. Our data suggests that VPS13C regulates lysosomal homeostasis through the regulation of phospho-Rab10 on the lysosomal membrane. Data represented as mean ± SEM; unpaired two-tailed *t* test (B, D, F, G, I, and J); **P < 0.01 (B, D, and J), ****P < 0.0001 (F, G, and I). Source data are available for this figure: SourceData F7.

Lysosomal enlargement in combination with impaired lysosomal function has been reported in various models of lysosomal storage and neurodegenerative disorders (de Araujo et al., 2020; Lie and Nixon, 2019). To probe for downstream effects of loss of VPS13C on neuronal lysosomes, we assessed various measures of lysosomal function, including hydrolytic activity of luminal hydrolases and acidification in control and VPS13C-deficient hiPSC-derived dopaminergic neurons. We observed significantly lower levels of mature cathepsin B and cathepsin D in VPS13C-deficient neurons compared with control neurons (Fig. 7, A–D). We further examined cathepsin B activity by using Magic Red Cathepsin B in live-cell confocal imaging. We found significantly fewer Magic Red-positive vesicles as well as a significantly lower mean fluorescence intensity of Magic Red-positive vesicles in VPS13C-deficient neurons, indicative of decreased cathepsin B activity (Fig. 7, E–G). Lastly, we evaluated whether loss of VPS13C affected lysosomal acidification. Interestingly, we also observed fewer LysoTracker-positive vesicles in VPS13C-deficient neurons as well as a significantly lower mean fluorescence intensity (Fig. 7, H–J), indicating altered lysosomal acidification. Together, these data suggest that enlarged lysosomes in VPS13C-deficient human dopaminergic neurons are also less functional with impaired hydrolytic activity and acidification.

**Figure S5.**
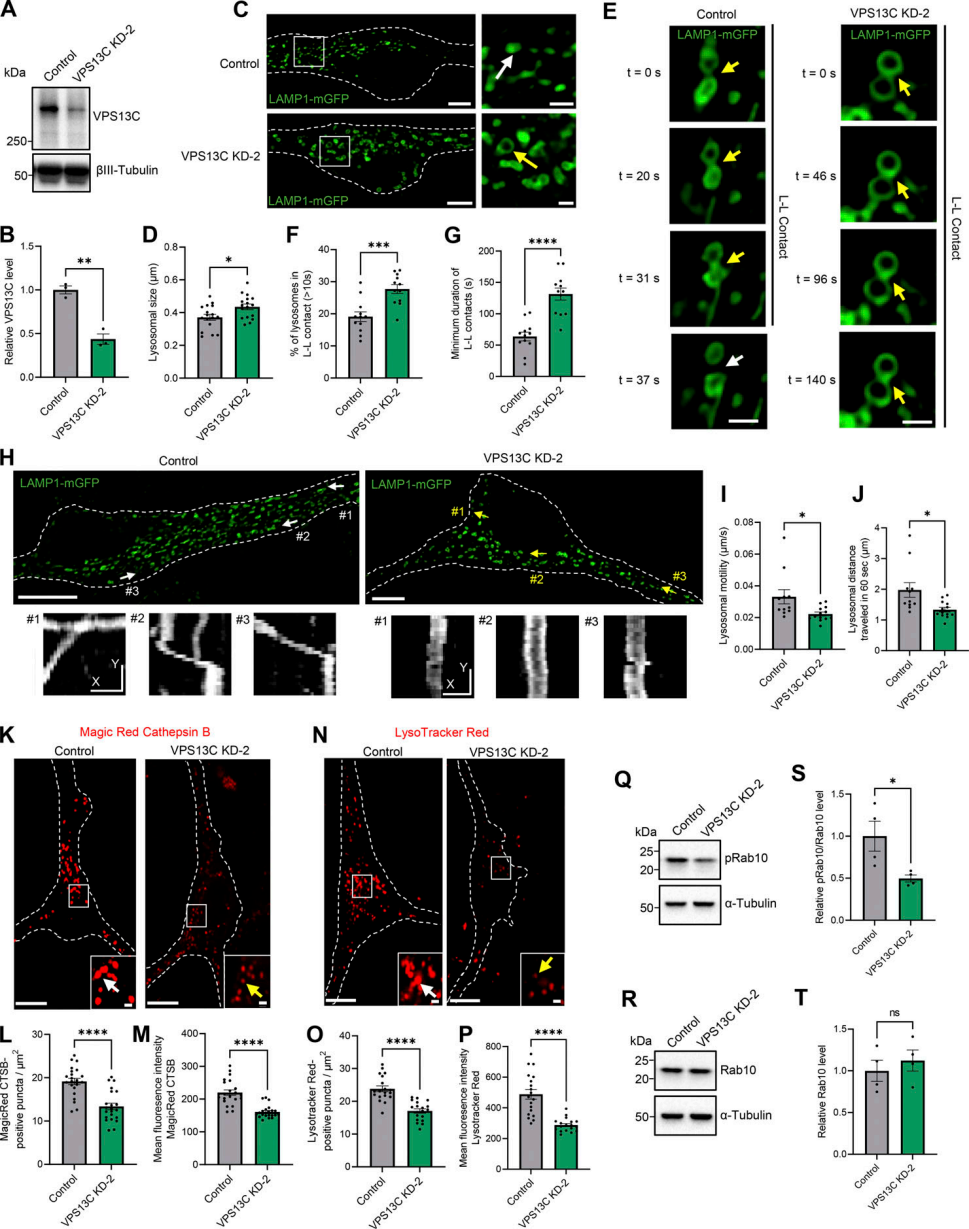
**Validation of lysosomal phenotypes with additional independent shRNA targeting VPS13C in iPSC-derived dopaminergic neurons. (A and B)** Representative immunoblot and quantification of VPS13C KD (KD-2) efficiency in hiPSC-derived dopaminergic neurons (day 70) after 14 days of control and VPS13C shRNA treatment (*N* = 3). **(C)** Representative live-cell confocal images of LAMP1-mGFP (green) -positive vesicles in control (upper) and VPS13C KD-2 (lower) neurons with insets showing enlarged lysosomes (yellow arrow) in VPS13C KD-2 condition in comparison with lysosomes in control condition (white arrow) (scale bar: 10 µm, inset: 1 µm). **(D)** Quantification of the average lysosomal size (*N* = 4, from *n* = 17 cells). **(E)** Time-lapse images of L-L contacts showing tethered lysosomes (yellow arrows) that untether at 37 s (white arrows) in control condition but remain tethered in VPS13C KD-2 neurons until 140 s (yellow arrow) (scale bar: 1 µm). **(F)** Quantification of the percentage of lysosomes in stable L-L contacts (≥10 s). **(G)** Quantification of the minimum duration of L-L contacts (*N* = 4, from *n* = 12 cells). **(H)** Representative live-cell confocal images showing LAMP1-positive vesicles (green) and examples of lysosomal motility demonstrated by kymographs (bottom panels) in control and VPS13C KD-2 neurons (scale bar: 10 µm, kymograph scale bar: x = 1 µm, y = 20 s). **(I and J)** Quantification of the average distance traveled by lysosomes in 60 s and the average lysosomal motility *N* = 4, from *n* = 11 cells (control) and *n* = 13 cells (VPS13C KD-2). **(K)** Representative live-cell confocal images of Magic Red cathepsin B staining in control neurons showing stronger cathepsin B intensity (white arrow) and in VPS13C KD-2 neurons showing reduced cathepsin B intensity (yellow arrow) Scale bar: 10 µm, inset: 1 µm. **(L and M)** Quantification of the number of Magic Red cathepsin B-positive puncta per cell area (L) and the mean fluorescence intensity of Magic Red cathepsin B-positive puncta (M) (*N* = 4, from *n* = 22 cells). **(N)** Representative live-cell confocal images of LysoTracker Red DND-99 staining in control neurons showing stronger LysoTracker intensity (white arrow) and in VPS13C KD-2 neurons showing reduced LysoTracker intensity (yellow arrows). Scale bar: 10 µm, inset: 1 µm. **(O and P)** Quantification of the number of LysoTracker Red-positive puncta per cell area (O) and the mean fluorescence intensity of LysoTracker Red-positive puncta (P) (*N* = 4, from *n* = 19 cells). **(Q–T)** Representative immunoblots and quantifications of phospho-Rab10 (pRab10) and total Rab10 protein levels from whole cell HEK-293 FT lysates (*N* = 4). Dashed lines in C, H, K, and N represent the outline of the cell. Data collected for the non-targeting control condition were used for the comparison between control and KD-1 in Figs. 1, 2, 3, 6, and 7, and for the comparison between control and KD-2 in Fig. S5. Data represented as mean ± SEM; unpaired two-tailed *t* test (B, D, F, G, I, J, L, M, O, P, S, and T); ns: not significant (T), *P < 0.05 (D, I, J, and S), **P < 0.01 (B), ***P < 0.001 (F), ****P < 0.0001 (G, L, M, O, and P). Source data are available for this figure: SourceData FS5.

Importantly, we used an additional independent shRNA targeting VPS13C in neurons (“VPS13C KD-2”) to further validate our findings that VPS13C regulates the dynamics and function of lysosomes as well as phospho-Rab10 levels (Fig. S5). We confirmed that VPS13C levels were significantly decreased by this additional shRNA targeting VPS13C in neurons (Fig. S5, A and B). In accordance with our prior results, KD of VPS13C in neurons with this independent shRNA resulted in significantly enlarged lysosomes (Fig. S5, C and D) that formed more stable inter-lysosomal contacts (Fig. S5, E–G) and had significantly decreased motility (Fig. S5, H–J). Importantly, using this independent shRNA targeting VPS13C, we validated that VPS13C KD altered lysosomal function by decreasing cathepsin B activity (Fig. S5, K–M) and impairing lysosomal acidification (Fig. S5, N and P). Finally, we confirmed that phospho-Rab10 levels were significantly decreased in VPS13C KD-2 cells (Fig. S5, Q–T). In conclusion, we have independently validated our initial findings that loss of VPS13C in neurons is an important regulator of lysosomal dynamics, function, and phospho-Rab10.

Together, these data suggest that under physiologic conditions, VPS13C regulates lysosomal homeostasis via stabilization of phospho-Rab10 on the lysosomal membrane. Without VPS13C, phospho-Rab10 dynamics on the lysosomal membrane are disrupted, resulting in enlarged lysosomes that are more tethered, less motile, and ultimately dysfunctional with impaired acidification and hydrolytic activity (Fig. 7 K), potentially contributing to the pathogenesis observed upon loss of VPS13C in PD.

## Discussion

Our data highlight a function of VPS13C in maintaining proper lysosomal dynamics and function by regulating the phospho-Rab10-mediated lysosomal stress response. Loss of VPS13C in hiPSC-derived dopaminergic neurons results in an increase in lysosomal size and inter-lysosomal contacts, which reduces lysosomal motility and increases perinuclear lysosomal positioning. Mechanistically, VPS13C interacts with Rab10 in a phospho-dependent manner on lysosomes, and loss of VPS13C disrupts the accumulation of phospho-Rab10 in response to lysosomal stress, thereby obstructing this critical pathway of lysosomal size regulation. Defective lysosomal dynamics and stress response in VPS13C-deficient dopaminergic neurons are further compounded by impaired lysosomal hydrolytic activity and acidification.

Our study adds to growing evidence for a role of VPS13C at the lysosome. Previous studies have suggested that VPS13C modulates several lysosomal functions in non-neuronal models, including lipid transfer at ER-lysosome contacts (Cai et al., 2022; Hancock-Cerutti et al., 2022; Kumar et al., 2018), lysosomal degradation of activated STING in response to mitochondrial DNA release (Hancock-Cerutti et al., 2022), and mitophagy (Lesage et al., 2016). Notably, our data indicate that VPS13C interacts with phospho-Rab10 and regulates lysosomal size via the phospho-Rab10-mediated lysosomal stress response. Under conditions of lysosomal stress, phospho-Rab10 accumulates onto damaged lysosomes, which activates downstream lysosomal content release to regulate stress-induced lysosomal enlargement (Eguchi et al., 2018). Interestingly, the PD-risk gene *LRRK2* has previously been shown to be involved in this lysosomal stress response pathway (Eguchi et al., 2018). Our data suggest that while lower phospho-Rab10 levels, like in VPS13C deficiency, lead to enlarged lysosomes, higher phospho-Rab10 levels like in LRRK PD lead to smaller lysosomes. Previous studies found that overexpression of various LRRK2 PD mutants decreased lysosomal size in comparison with LRRK2 WT (Eguchi et al., 2018), and decreased average lysosomal size was also observed in LRRK2 G2019S knock-in neurons from primary cultures (Schapansky et al., 2018). This indicates that tight regulation of Rab10 phosphorylation may be important in PD and that either increasing phospho-Rab10 with LRRK2 mutants or decreasing phospho-Rab10 with VPS13C deficiency may lead to misregulation of this pathway and contribute to disease pathophysiology.

Additionally, several studies have reported the recruitment of LRRK2 to stressed and damaged lysosomes to promote lysosomal membrane repair via the ESCRT-III–mediated repair pathway (Bonet-Ponce et al., 2020; Eguchi et al., 2018; Herbst et al., 2020; Radulovic and Stenmark, 2020). While current studies suggest the involvement of LRRK2 in this pathway, it will be interesting to investigate whether Rab10 and VPS13C also participate in this lysosomal repair mechanism. Importantly, given VPS13C’s reported function as a lipid transport protein (Cai et al., 2022; Hancock-Cerutti et al., 2022; Kumar et al., 2018), the lipid transport function of VPS13C may also contribute to its regulation of lysosomal dynamics and function in iPSC-derived dopaminergic neurons. Moreover, future studies aimed at mapping the interaction site between VPS13C and Rab10 will further elucidate their phospho-dependent interaction and how this interaction regulates lysosomal homeostasis.

Defective inter-organelle contacts have been observed in various models of neurodegenerative diseases including PD (Cisneros et al., 2022; Kim et al., 2021, 2022; Peng et al., 2023; Wilson and Metzakopian, 2021). Our work demonstrates that VPS13C-deficient human dopaminergic neurons exhibit increased inter-lysosomal tethering, which impairs lysosomal motility and leads to more perinuclear positioning. Inter-lysosomal tethers were recently found to be modulated by mitochondria and act as essential regulators of lysosomal network dynamics (Wong et al., 2022). In accordance with our findings, lysosomes in inter-lysosomal contacts were shown to have significantly decreased lysosomal motility and increased perinuclear localization. Importantly, mutations in microtubule-dependent motor adaptors did not disrupt inter-lysosomal contact formation or contact tethering dynamics (Wong et al., 2022), suggesting that while misregulation of lysosomal positioning is not sufficient to alter lysosomal contact dynamics, disruption of lysosomal tethering dynamics is able to misregulate both lysosomal motility and distribution. Accordingly, we observed impaired lysosomal motility and distribution in VPS13C-deficient neurons secondary to increased inter-lysosomal contacts.

We further show that VPS13C modulates lysosomal contacts with the ER and mitochondria. In agreement with previous studies, we found that VPS13C preferentially localizes to ER-lysosome contacts (Cai et al., 2022; Kumar et al., 2018). Loss of VPS13C in neurons altered ER-lysosome contact dynamics by significantly increasing ER-lysosome contact formation. Interestingly, we also found that VPS13C can regulate mitochondria-lysosome contact site formation and duration, suggesting a potential secondary effect on mitochondrial dynamics and function. A previous report demonstrated that VPS13C KD can lead to abnormal mitochondria morphology and mitochondrial dysfunction (Lesage et al., 2016). Together, our data support an additional function of VPS13C in regulating inter-organelle contacts involving lysosomes.

Lysosomal dysfunction has been implicated as a potential converging mechanism across various forms of PD (Navarro-Romero et al., 2020; Wallings et al., 2019). VPS13C was recently identified as a genetic cause of early-onset PD (Darvish et al., 2018; Gu et al., 2020; Hopfner et al., 2020; Jansen et al., 2017; Lesage et al., 2016; Nalls et al., 2014, 2019; Pan et al., 2023; Schormair et al., 2018; Smolders et al., 2021), yet how loss-of-function of VPS13C leads to PD remains uncertain. Our study suggests that VPS13C contributes to PD pathogenesis through dysregulation of several lysosomal pathways including inter-organelle contacts and the Rab10-mediated lysosomal stress response, which ultimately led to lysosomal dysfunction. Proper lysosomal network dynamics and function are of particular importance to post-mitotic cells such as neurons, which require efficient bidirectional lysosomal transport mechanisms and preserved lysosomal degradative capacity to support neuronal health (Roney et al., 2022). Future studies investigating how these various lysosomal pathways intersect and contribute to neuronal dyshomeostasis will further shed light on the pathophysiology of PD and other neurodegenerative diseases presenting with lysosomal dysfunction.

## Materials and methods

### Cell culture and transfection

HEK-293 FT and COS7 cells were cultured in Dulbecco’s Modified Eagle Medium (DMEM, 11995073; Invitrogen) supplemented with 10% (vol/vol) heat-inactivated fetal bovine serum (HI-FBS, 100-106; Gemini Bio-Products), 100 U/ml penicillin (pen), and 100 µg/ml streptomycin (strep) (15140-122; Invitrogen) at 37°C and 5% CO_2_. HEK-293 FT cells were plated according to the following seeding densities: 180,000/well (12-well), 480,000/well (6-well), 2.7 million/dish (10 cm), and 7.8 million/dish (15 cm). COS7 cells were plated at 150,000/well in 36-mm live-cell chambers with a glass bottom. Cells were transfected with X-tremeGENE HP transfection reagent (6366236001; Sigma-Aldrich/Roche) at a 3:1 ratio according to the manufacturer’s protocol for 24–48 h, or alternatively, cells were transfected with Lipofectamine 2000 (11668-019; Invitrogen) according to the manufacturer’s protocol.

### iPSC culture and midbrain dopaminergic neuron differentiation

hiPSC lines were obtained from the Northwestern University Biorepository and Coriell Institute. hiPSCs were generated from skin fibroblasts using retroviral expression of OCT4, SOX2, c-Myc, and KLF4 (A16517; CTS Cyto Tune-iPS 2.0 Sendai Reprogramming Kit; Invitrogen) (Takahashi et al., 2007). Healthy control iPSCs were cultured using mTeSR Plus Basal Medium (100-0276; Stem Cell Technologies) supplemented with mTesR Plus 5x Supplement (Stem Cell Technologies) on Cultrex-coated (3434-010-02; R&D Systems) 6-well plates at 37°C and 5% CO_2_. iPSC colonies were manually passaged every 6 days. Cells were routinely tested for mycoplasma contamination (Venor GeM Mycoplasma Detection Kit, MP0025; Sigma-Aldrich).

Dopaminergic neuron differentiation was performed using previously published protocols (Kriks et al., 2011) with minor modifications. Cells were passaged as 1–2 mm chunks on day 13 and plated on poly-D-lysine (PDL)/laminin-coated 10-cm dishes. On day 25, cells were treated with Accutase (A6964; Sigma-Aldrich) to achieve single cells and plated on PDL/laminin-coated cell culture dishes and maintained in neurobasal medium (21103049; Gibco) supplemented with pen/strep, L-Glutamine, Neurocult SM1 (05711BD; Stem Cell Technologies), BDNF (248-BDB-050/CF; R&D Systems), ascorbid acid (A5960; Sigma-Aldrich), GDNF (212-GD-050; R&D Systems), TGF-β3 (8420-B3-025; R&D Systems), Dibutryl-cAMP (BML-CN125; Enzo Lifesciences), and DAPT (2634/10; R&D Systems) until day 40 with half media changes every 3 days. Single cells were plated on day 25 as follows: 600,000/well (12-well), 1.2 million/well (6-well), and 125,000/well (four-chamber live-cell imaging dish with glass bottom). Neuronal growth factors were withdrawn at day 40 and dopaminergic neurons were maintained in neurobasal medium with SM1 supplement. For quality control purposes, each differentiation was tested via immunofluorescence for βIII-Tubulin and TH on days 50–55. All other neuronal experiments were conducted on day 70. Differentiated dopaminergic neurons were transduced with lentiviruses as indicated.

### Plasmids

LAMP1-mGFP (34831; Addgene) was a gift from Esteban Dell-Angelica (Falcón-Pérez et al., 2005), LAMP1-RFP (1817; Addgene) was a gift from Walther Mothes (Sherer et al., 2003), BFP-KDEL (49150; Addgene) was a gift from Gia Voeltz (Friedman et al., 2011), Halo-KDEL (124316; Addgene) was a gift from Jin Wang (Jiang et al., 2019), mApple-TOMM20-N-10 (54955; Addgene) was a gift from Michael Davidson, LAMP1-RFP-3xHA (102932; Addgene) was a gift from David Sabatini (Abu-Remaileh et al., 2017), pEGFP-N1-Flag (60360; Addgene) was a gift from Patrick Calsou (Britton et al., 2014), HA-GFP-Myc (137763; Addgene) was a gift from Carol Mercer (Stefely et al., 2020), pER4, psPAX2 (12260; Addgene) was a gift from Didier Trono, pLP3 (K497500; Invitrogen), VPS13C-Myc-His was a gift from Antonio Velayos-Baeza (Yang et al., 2016), VPS13C^mClover3 (118760; Addgene) was a gift from Pietro de Camilli (Kumar et al., 2018), and eGFP-Rab10 (49472; Addgene) was a gift from Marci Scidmore (Rzomp et al., 2003). Rab10 point mutations (T73E and T73A) were introduced using Q5-site-directed mutagenesis (New England Biolabs). Plasmids of eGFP-Rab10 WT/TE/TA were subcloned into pmCherry-C1 vector. The following plasmids have been created with standard cloning methods: pER4-LAMP1-mGFP, pER4-Halo-KDEL, pER4-mApple-TOMM20-N-10, and pCMV-LAMP1-RFP-3xHA.

### Generation and transduction of lentiviral constructs

Lentiviral shRNA constructs for non-targeting control and VPS13C were obtained from Horizon Discovery/Sigma-Aldrich (RHS6848, TRCN0000232492 [KD], TRCN0000150922 [KD-2]). One non-targeting control construct was used for the comparison to KD-1 and KD-2. Constructs for LAMP1-mGFP and KDEL-Halo were obtained from Addgene and were subcloned into the pER4 lentiviral expression vector using standard cloning methods via NheI/NotI cutting sites. Lentiviral targeting vectors along with helper plasmids PAX2 (Addgene) and pLP3 (Invitrogen) were transfected into HEK-293 FT cells using X-tremeGENE transfection reagent. Lentivirus-containing supernatant was collected, filtered with 0.45-µm PES filter (Celltreat), and concentrated using Lenti-X-concentrator (631232; Clontech) 48 h after transfection. The supernatant mix was incubated overnight at 4°C. Concentrated virus was aliquoted and stored at -80°C. The quantification of retroviral antigens was performed using the HIV-1 p24 Antigen ELISA Kit (22-156-700; Thermo Fisher Scientific).

Differentiated dopaminergic neurons were transduced with concentrated lentivirus for non-targeting control shRNA or VPS13C-specific shRNA (KD versus KD-2) at day 56 with MOI 2 and incubated for 14 days.

For live-cell imaging microscopy, differentiated neurons were transduced with lentiviruses LAMP1-mGFP, mApple-TOMM20, and KDEL-Halo at MOI 5 for 5–7 days prior to imaging.

### Antibodies

Primary antibodies for western blotting were VPS13C (HPA043507, 1:500; Sigma-Aldrich, 28676-1-AP, 1:800; Proteintech), phosphoRab10-T73 (230261, 1:500; Abcam), Rab10 (8127, 1:1,000; Cell Signaling), βIII-Tubulin (4466S, 1:4,000; Biolegend), TH (657012, 1:2,000; Millipore), GAPDH (2,118, 1:2,000; Millipore), α-Tubulin (5168, 1:20,000; Sigma-Aldrich), LAMP2 (H4B4, 1:1,000; DSHB), GCase (G4171, 1:1,000; Sigma-Aldrich), Rab7 (137029, 1:1,000; Abcam), HA (3724, 1:2,000; Cell Signaling), Calnexin (1:1,000; Cell Signaling), TOM20 (612278, 1:1,000; BD Biosciences), PEX5 (83020S, 1:500, Cell Signaling), GFP (1544, 1:2,000; Sigma-Aldrich), Myc (2278, 1:2,000; Cell Signaling), cathepsin B (AF953, 1:2,000; R&D systems), cathepsin D (6487, 1:1,000; Santa Cruz), LRRK2 (ab133474, 1:500; Abcam), LRRK2-S935 (ab133450, 1:500; Abcam), EHBP1 (17637-1-AP, 1:1,000; Proteintech), and PPM1H (PA5-26102, 1:1,000; Invitrogen).

Primary antibodies for immunofluorescence were βIII-Tubulin (4466S, 1:300; Biolegends), TH (657012, 1:300; Millipore), and LAMP1 (sc-20011, 1:300; Santa Cruz).

Primary antibodies for PLA were VPS13C (28676-1-AP, 1:100; Proteintech) and Rab10 (ab104859, 1:100; Abcam).

#### Secondary antibodies

HRP-conjugated secondary antibodies were obtained from Jackson Immuno Research Laboratories and used at 1:5,000 for western blot analysis (Goat-anti-Mouse-HRP, Goat-anti-Rabbit-HRP, and Bovine-anti-Goat-HRP); and Alexa-Fluor–conjugated secondary antibodies for immunofluorescence were obtained from Invitrogen and used at 1:500 (Goat-anti-Mouse-AlexaFluor568, Goat-anti-Rabbit-AlexaFluor488).

### Treatment conditions

HEK-293 FT cells were treated with CQ as previously described (Eguchi et al., 2018). Briefly, HEK-293 FT cells were plated and cultured for 30 h before treatment with CQ (C6628; Sigma-Aldrich). CQ was diluted in Opti-MEM and carefully added to the culturing media at a final concentration of 100 µM. Alternatively, HEK-293 FT cells were treated with LLOMe (16008; Cayman) at a final concentration of 500 µM. Incubation time was as indicated in the figure legend. For co-IP and PLA experiments, HEK-293 FT cells were treated with MLi-2 (5756/10; R&D) for 16 h overnight and treated for an additional 2 h with fresh MLi-2 prior to the experiment at a final concentration of 200 nM.

For live-cell imaging microscopy, differentiated dopaminergic neurons were incubated with LysoTracker Red DND-99 (L7528; Thermo Fisher Scientific) (50 nM), Magic Red cathepsin B (ICT937; BioRad) (1:2,000), or Calcein AM-488 (20 nM) for 30 min in fresh culture media. For the visualization of Halo-tag, cells were incubated with Janelia Fluor Halo tag ligand-646 (GA1120; Promega) (1:3,333) for 30 min. Cells were imaged after three quick washes in fresh culturing media. Imaged cells were randomly selected based on Calcein-488 staining to achieve blinding of the investigator.

### Immunofluorescence

For the immunofluorescence of hiPSC-derived dopaminergic neurons, cells were cultured on nitric acid–treated and PDL/laminin (Sigma-Aldrich) -coated coverslips until the desired day of staining. Cells were briefly washed in 1xPBS and fixed using 4% paraformaldehyde in 1xPBS for 20 min at room temperature. Cells were washed three times in ice-cold 1xPBS and then permeabilized using permeabilization buffer (10% HI-FBS in 1xPBS, 0.1% Saponin) for 30 min at room temperature. Cells were then incubated with primary antibodies diluted in permeabilization buffer overnight at 4°C. Coverslips were washed three times in 1xPBS and incubated with corresponding secondary antibodies for 1 h at room temperature. Samples were washed three times with 1xPBS and finally mounted onto SuperfrostPlus microscope slides (12-550-15; Fisherbrand) using DAPI-Fluoromount-G (0100-20; Southern Biotech). Image stacks were obtained using a Nikon W1 spinning disc microscope with 60× OIL objective at z-steps of 0.5 µm for TH imaging and 0.2 µm for LAMP1 imaging. Imaged cells were randomly selected based on DAPI staining. For the analysis of the percentage of TH-positive neurons, multiple Z-stack confocal images were obtained of each culture condition and subsequently segmented for both TH and DAPI channels, followed by analysis of the number of TH- and DAPI-positive neurons in every Z-stack. At least 100 neurons were quantified per biological replicate. Representative images for LAMP1 staining in dopaminergic neurons were processed via 3D deconvolution (automatic mode) in Nikon Elements software 5.3.

### Generation of protein lysates and SDS-PAGE/western blotting

Cells were briefly washed and then scraped in ice-cold 1xPBS and centrifugated at 300 × *g* for 5 min at 4°C. Cell pellets were lysed in 1x radioimmunoprecipitation assay lysis buffer (Boston Bio products) with protease inhibitor cocktail (cOmplete, 1183617001; Millipore) and subsequently sonicated (3 × 10 s, amplitude 30) to ensure full lysis and incubated on ice for up to 1 h. Samples were centrifugated at 20,000 × *g* for 10 min and the supernatant was used to determine the total protein concentration using the Bicinchoninic Acid Assay (BCA, 23225; Thermo Fisher Scientific). Lysates were diluted in 4x Laemmli buffer (1610747; BioRad) containing β-mercaptoethanol and boiled at 95°C for 5 min. For the detection of phosphoproteins, cells were directly lysed in 1x Laemmli buffer without bromophenol blue or β-mercaptoethanol (32.9 mM Tris-HCl, pH 6.8, 1.05% [wt/vol] SDS, 13.15% [wt/vol] glycerol) for 10 min and boiled at 100°C for 10 min. Boiled protein samples were separated using precasted 4–12% or 4–20% Tris-glycine gels (Invitrogen) and subsequently transferred either via semidry method on nitrocellulose membrane for 10 min at 2.5 A (Trans-Blot Turbo; BioRad) or for larger proteins via wet-transfer method (Criterion, BioRad) on PVDF membrane at 0.8 A for 2 h on ice. Membranes were blocked with 5% non-fat dry milk in 1xTBS-T for 1 h at room temperature and incubated overnight at 4°C with the desired primary antibody diluted in 5% BSA in 1xTBS-T. Membranes were washed three times with 1xTBS-T at room temperature and incubated for 1 h with the corresponding HRP-linked secondary antibody diluted in 5% milk in TBS-T. Membranes were washed again three times in 1xTBS-T and quickly rinsed in water before imaging on the ChemiDoc XRS+ (BioRad). Analysis and quantification of protein bands were performed using ImageLab 6.1.

### co-IP

HEK-293 FT cells were transfected with X-tremeGENE (Roche) at a 3:1 ratio (transfection reagent:DNA) and incubated for 24–48 h prior to IP. Cells were briefly washed, scraped, and centrifugated in ice-cold 1xPBS and subsequently lysed in AP-MS buffer (10 mM Tris/Cl, pH 7.5, 150 mM NaCl, 10% glycerol, 0.5 mM EDTA, 0.5% Nonidet P40) with protease inhibitor cocktail (cOmplete; Millipore) by sonification and incubated on ice for 1 h. Cell lysates were centrifugated at 20,000 × *g* for 10 min at 4°C. The protein concentration of the supernatant was determined by BCA and equal protein amounts were used for IP. Magnetic c-Myc (88843; Thermo Fisher Scientific) or GFP-Trap Agarose (gta-20; ChromoTek) beads were blocked with 5% BSA in AP-MS buffer (2 h, 4°C) prior to IP. The protein lysates were incubated with the blocked beads for either 2 h (GFP-Trap) or overnight (magnetic c-Myc) while rotating at 4°C. Following incubation, beads were washed four times with AP-MS buffer for each 5 min while rotating. Immunoprecipitated proteins were eluted from the beads by adding 2x Laemmli buffer and boiling at 55°C for 25 min. Samples were then subjected to SDS-PAGE and western blot analysis. Alternatively, immunoprecipitated proteins were analyzed by MS.

### AP-MS

For IP followed by MS analysis, HEK-293 T cells were transfected with cDNA for GFP-Rab10 WT, phosphomimetic GFP-Rab10 T73E (TE), phosphodeficient GFP-Rab10 T73A (TA), or mock-GFP, which served as a control. Proteins were immunoprecipitated with GFP-Trap beads as described above. Following incubation, beads were collected by centrifugation (2,500 × *g*, 2 min, 4°C) and washed three times with AP-MS buffer and two times with AP-MS without glycerol and detergent. Next, beads were incubated with elution buffer I (50 mM Tris/Cl, pH 7.5, 2 M urea, 5 µg/ml sequencing grade modified trypsin, 1 mM DTT) for 30 min at 30°C and centrifuged (2.500 × *g*, 2 min). Supernatants from each sample were collected. Beads were resuspended in elution buffer II (50 mM Tris/Cl, pH 7.5, 2 M urea, and 5 mM iodoacetamide). After centrifugation, beads were discarded, and supernatants from elution I and II were combined and incubated at 32°C overnight.

The next steps were performed as previously described (Donkervoort et al., 2021; Nguyen and Krainc, 2018). Briefly, the precipitated protein pellets were solubilized in 100 µl of 8 M urea for 30 min, 100 µl of 0.2% ProteaseMAX (Promega) was added, and the mixture was incubated for an additional 2 h. The protein extracts were reduced and alkylated as described previously (Chen et al., 2008), followed by the addition of 300 µl of 50 mM ammonium bicarbonate, 5 µl of 1% ProteaseMAX, and 20 µg of sequence-grade trypsin (Promega). Samples were digested overnight in a 37°C thermomixer (Eppendorf).

For Orbitrap Fusion Tribrid MS analysis, the tryptic peptides were purified with Pierce C18 spin columns and fractionated with increasing ACN concentration (15, 20, 30, 40, 60, and 70%). 3 µg of each fraction were auto-sampler loaded with a Thermo Fisher Scientific EASY nLC 1000 UPLC pump onto a vented Acclaim Pepmap 100, 75 µm × 2 cm, nanoViper trap column coupled to a nanoViper analytical column (164570, 3 µm, 100 Å, C18, 0.075 mm, 500 mm; Thermo Fisher Scientific) with stainless steel emitter tip assembled on the Nanospray Flex Ion Source with spray voltage of 2,000 V. Buffer A contained 94.785% H_2_O with 5% ACN and 0.125% FA, and buffer B contained 99.875% ACN with 0.125% FA. The chromatographic run was for 4 h in total with the following profile: 0–7% for 7 min, 10% for 6 min, 25% for 160 min, 33% for 40 min, 50% for 7 min, 95% for 5 min, and 95% again for 15 min. Additional MS parameters include ion transfer tube temp = 300°C, Easy-IC internal mass calibration, default charge state = 2, and cycle time = 3 s. Detector type set to Orbitrap, with 60 K resolution, with wide quad isolation, mass range = normal, scan range = 300–1,500 m/z, max injection time = 50 ms, AGC target = 200,000, microscans = 1, S-lens RF level = 60, without source fragmentation, and datatype = positive and centroid. MIPS was set as on, included charge states = 2–6 (reject unassigned). Dynamic exclusion enabled with n = 1 for 30 and 45 s exclusion duration at 10 ppm for high and low. Precursor selection decision = most intense, top 20, isolation window = 1.6, scan range = auto normal, first mass = 110, collision energy 30%, CID, detector type = ion trap, orbitrap resolution = 30 K, IT scan rate = rapid, max injection time = 75 ms, AGC target = 10.000, Q = 0.25, inject ions for all available parallelizable time.

### Tandem mass spectra analysis

Peptide spectral files from pooled samples or biological replicates were combined for database searching. Spectrum raw files were extracted into MS1 and MS2 files using in-house program RawXtractor or RawConcerter (http://fields.scripps.edu/downloads.php) (He et al., 2015), and the tandem mass spectra were searched against UniProt human database (downloaded on UniProt_Human_proteome_cont_03-25-2014) and matched to sequences using the ProLuCID/SEQUEST algorithm (ProLuCID version 3.1) (Eng et al., 1994; Xu et al., 2006).

The search space included all fully and half-tryptic peptide candidates that fell within the mass tolerance window with no miscleavage constraint, assembled, and filtered with DTASelect2 (version 2.1.3) (Cociorva et al., 2007; Tabb et al., 2002) through Integrated Proteomics Pipeline (IP2 version 3, Integrated Proteomics Applications, https://www.manula.com/manuals/ip2/ip2/1/en/topic/system). To estimate peptide probabilities and false-discovery rates (FDR) accurately, we used a target/decoy database containing the reversed sequences of all the proteins appended to the target database (Peng et al., 2003). Each protein identified was required to have a minimum of one peptide of a minimal length of six amino acid residues; however, this peptide had to be an excellent match with an FDR < 0.001 and at least one excellent peptide match. After the peptide/spectrum matches were filtered, we estimated that the protein FDRs were ≤1% for each sample analysis.

### LysoIP

LysoIP was performed as previously described (Abu-Remaileh et al., 2017). Briefly, HEK-293 FT cells were plated on 15-cm dishes (see plating density from above), transfected with LAMP1-RFP-3xHA, and incubated for 48 h after transfection. Cells were washed in ice-cold 1xPBS and scraped in KBPS buffer (136 mM KCl, 10 mM KH_2_PO_4_, pH 7.25) and supplemented with protease inhibitor cocktail. The cell suspension was centrifugated at 1,000 × *g* for 2 min at 4°C. Cells were resuspended in KPBS buffer and homogenized gently using a 23 G syringe (BD Integra, 14-823-52; Thermo Fisher Scientific). Homogenized cells were centrifugated at 1,000 × *g* for 3 min at 4°C. The supernatant was applied to 100 µl magnetic anti-HA beads (88837; Thermo Fisher Scientific) that were prewashed and preblocked in KPBS buffer with 5% BSA. Samples were incubated for 15 min while rotating at 4°C. After the incubation, the beads were washed five times in KPBS buffer. Samples were eluted in 100 µl 1x Laemmli buffer without bromophenol blue or β-mercaptoethanol and boiled at 95°C for 10 min. Samples were quantified via BCA and equal protein amounts were used for Western Blot analysis.

### Live-cell confocal microscopy

Confocal live-cell imaging was performed using a Nikon W1 Spinning Disk microscope with a 100×-oil objective (TIRF 100x 1.49 NA; Nikon Plan Apo). Dopaminergic neurons (day 70) were cultured and transduced or stained as described above and imaged in four-chamber glass-bottom dishes (D35C4-20-1.5-N; Cellvis) in a temperature-controlled (37°C) and a humidified chamber with 5% CO_2_. Images were acquired in single-camera mode with 500-ms exposure time. Cells were imaged at 1 frame every 2 s for 3 min total. For 3D live-cell imaging, cells were imaged as stated above with z-steps of 0.2 µm. All live-cell confocal images for analysis of Rab10 and VPS13C localization in live COS7 cells were acquired on a Nikon A1R laser scanning confocal microscope with GaAsp detectors using a Plan Apo λ 100x 1.45 NA oil immersion objective (Nikon) using NIS-Elements (Nikon). Live cells were imaged in a temperature-controlled chamber (37 °C) at 5% CO_2_ at one frame every 2–3 s. Dual-color videos were acquired as consecutive green-red images and tricolor videos were acquired as consecutive green-red-blue images.

### Image processing and analysis

Confocal live-cell images were processed and analyzed using NIS-Elements 5.3 software (Nikon). To correct for photobleaching, time-lapsed images were corrected using intensity equalization over time and further processed using Denoise AI and the 2D deconvolution module (automatic mode). Data were analyzed from the somato-dendritic region of iPSC-derived dopaminergic neurons at day 70.

For the lysosomal size analysis, LAMP1-mGFP signal was thresholded. The percentage of cell area occupied by lysosomes was calculated by measuring the area of the LAMP1-mGFP binary divided by the cell area. The average lysosomal size was measured per object. Only objects between 0.1 and 2 µm were considered.

Stable inter-lysosomal contacts were defined as two lysosomes that stayed in contact with one another for at least 10 s (Wong et al., 2018, 2022). The percentage of lysosomes in contact with other lysosomes was quantified as the percentage of lysosomes that were in contact with each other at t = 0 s of the time-lapse video and stayed in contact for at least 10 s. For the analysis of the minimum duration of L–L contacts, only contacts that had formed at t = 0 s were considered. At random, five of those contacts per video and cell were monitored over time until termination and dissociation of the two organelles or until the end of the video (3 min; 180 s). Any contact that lasted throughout the entire video was categorized as 180 s (Wong et al., 2022).

The motility of lysosomes was measured using the tracking module in NIS-Elements. Only lysosomes that were tracked for 60 s were considered for the analysis. The distance traveled by those lysosomes within 60 s was measured as well as the average lysosomal motility.

Lysosomal distance analysis was performed using the General Analyzer module in NIS-Elements. The distance of every single lysosome to the nuclear membrane within the somatodendritic region of iPSC-derived dopaminergic neurons was measured. The nuclear membrane was manually added as a binary and converted into a color component via Halo-KDEL staining. The green channel for LAMP1-mGFP was thresholded. Thresholding quality was improved using rolling ball background correction. Only objects with a size between 0.1 and 2 µm were considered. The perinuclear region was defined as <7 µm from the nuclear membrane, and distal lysosomes were characterized as all lysosomes further than ≥7 µm from the nuclear membrane.

The percentage of lysosome-ER contacts was calculated from the number of lysosomes overlapping with ER divided by the total number of lysosomes per cell. For analysis of lysosome-ER contact dynamics, the signals of LAMP1-mGFP and Halo-KDEL were thresholded to generate binaries for both channels, and the overlap of the LAMP1-mGFP and Halo-KDEL binaries was generated. The average duration of lysosome-ER contacts was measured using the Tracking module in NIS-Elements. Only objects in the lysosome-ER overlap binary with stable tracks lasting >10 s were used to calculate the average duration of lysosome-ER contacts. The percentage of cell area occupied by the ER was calculated by measuring the area of the Halo-KDEL binary divided by the total cell area.

The General Analyzer tool was used for the analysis of Magic Red cathepsin B and LysoTracker Red DND-99 staining. Briefly, background correction was performed using rolling ball correction and Magic Red and LysoTracker signal was thresholded and filtered for all objects between 0.1 and 2 µm. The number of positive puncta (MagicRed or LysoTracker Red) was calculated by the number of puncta divided by the cell area, and the mean fluorescence intensity was measured per thresholded object.

For analysis of Rab10 and VPS13C localization in live cells, images were analyzed in NIS-Elements. The percentage of lysosomes that were positive for Rab10 (WT/TE/TA) per cell was analyzed as the percentage of LAMP1-mGFP–positive vesicles in a subregion of the cell that were positive for mCherry-Rab10 WT, mCherry-Rab10 TE, or mCherry-Rab10 TA. The percentage of cells that were ER-positive for Rab10 (WT/TE/TA) per experiment was analyzed as the percentage of cells with BFP-KDEL, which was positive for mCherry-Rab10 WT, mCherry-Rab10 TE, or mCherry-Rab10 TA. The percentage of VPS13C vesicles that were positive for Rab10 (WT/TE/TA) per cell was analyzed as the percentage of VPS13C^mClover3-positive vesicles in a subregion of the cell that was positive for mCherry-Rab10 WT, mCherry-Rab10 TE, or mCherry-Rab10 TA.

The number of VPS13C vesicles per cell was analyzed as the total number of VPS13C^mClover3-positive vesicles in a cell expressing LAMP1-RFP (control), mCherry-Rab10 WT, mCherry-Rab10 TE, or mCherry-Rab10 TA.

The number of Rab10 vesicles per cell was analyzed as the total number of mCherry-Rab10 WT-positive vesicles in a cell expressing LAMP1-mGFP (control) or VPS13C^mClover3.

### PLA

The interaction between VPS13C and Rab10 was validated via PLA. VPS13C knockdown and non-targeting control HEK-293 FT cells were plated on PDL-coated coverslips and cultured for 2 days before the assay. The day after plating, HEK-293 FT cells were treated with 200 nM MLi-2 or DMSO overnight. Cells were treated with another dose of 200 nM MLi-2 2 h before the assay. Dopaminergic neurons infected with lentivirus for non-targeting control or VPS13C-specific shRNA were cultured for 2 wk before the assay. Cells were fixed with 4% PFA for 20 min and permeabilized with 0.1% Saponin for 1 h at room temperature. The PLA was performed using the Duolink In Situ Red Starter Kit Mouse/Rabbit (DUO92101; Sigma-Aldrich) following the manufacturer’s instructions with minor modifications. Briefly, permeabilized cells were blocked with Duolink blocking solution for 2 h at 37°C and incubated with mouse anti-Rab10 (ab104859, 1:100; Abcam) and rabbit anti-VPS13C (28676-1-AP, 1:100; Proteintech) antibodies overnight at 4°C. The next day, cells were washed and incubated with Duolink anti-mouse-MINUS and anti-rabbit PLUS probes for 1 h, followed by ligation for 45 min and amplification for 3 h at 37°C. Cells were mounted using mounting medium with DAPI. PLA-Red signal was imaged on a Leica confocal microscope with a 63x oil objective. Imaged cells were randomly selected based on DAPI staining. At least 24 stacks with z-series at 0.5 μm per section were acquired from three to four biological replicates in each condition. The number and total area of PLA puncta were quantified across maximum intensity projections of each stack using Image J and then divided by the number of cells (DAPI^+^ nuclei). All data were presented as fold changes relative to the mean of the corresponding control.

### Statistical analysis, graphing, and figure assembly

Data were analyzed using unpaired- or paired two-tailed Student’s *t* test for two dataset comparisons or one-way ANOVA with Tukey’s multiple comparisons test for three or more dataset comparisons (see figure legends for details). Data distribution was assumed to be normal, but this was not formally tested. Statistical analysis and data graphing were performed with GraphPad Prism 9.0 software. All data are presented as means ± SEM with P values ≤ 0.05 considered significant. Generated data for the non-targeting control condition were used for the comparison between control and KD-1 in Figs. 1, 2, 3, 6, and 7, and for the comparison between control and KD-2 in Fig. S5. For the analysis of AP-MS data sets, MetabloAnalyst 5.0 was used, P value ≤ 0.01 was considered significant. All statistical analysis was performed from at least three independent biological experiments (see figure legends for details). Images and videos from live-cell confocal imaging were analyzed using NIS-Elements 5.3 (Nikon). Immunofluorescence images were analyzed using ImageJ (National Institutes of Health). All figures were assembled in Microsoft PowerPoint and Adobe Illustrator. Schematic figures were created with Microsoft PowerPoint and https://BioRender.com.

### Online supplemental material

Fig. S1 shows the differentiation process of hiPSCs into dopaminergic neurons and lentiviral shRNA treatment for VPS13C KD, characterization of control and VPS13C KD dopaminergic neurons, and endogenous LAMP1 staining in dopaminergic neurons. Fig. S2 shows the analysis of ER-lysosome and mitochondria-lysosome contact site formation and duration in iPSC-derived dopaminergic neurons. Fig. S3 shows localization studies of VPS13C-mClover and analysis of VPS13C-positive vesicles with Rab10 and vice versa. Fig. S3 also shows LysoIP experiments at baseline and upon treatment with lysosomal stressor CQ. Fig. S4 shows LysoIP approaches specifically looking at LRRK2 and LRRK2-S935 protein levels at baseline and upon lysosomal stressor CQ, as well as LAMP1-mGFP confocal imaging in LRRK2 PD-mutant (R1441G) and isogenic control neurons. Fig. S5 shows extensive validation of lysosomal dynamics and function in iPSC-derived dopaminergic neurons with an additional independent shRNA targeting VPS13C (KD-2) in comparison with control neurons. Data S1 contains the raw and analyzed dataset from the AP-MS screen for Rab10 interactors, and Videos 1 and 2 show lysosomal motility in control (Video 1) and VPS13C KD (Video 2) dopaminergic neurons.

## Supplementary Material

Data S1 is a dataset from unbiased affinity purification following mass spectrometry screen.

SourceData F1 is the source file for Fig. 1.

SourceData F4 is the source file for Fig. 4.

SourceData F5 is the source file for Fig. 5.

SourceData F6 is the source file for Fig. 6.

SourceData F7 is the source file for Fig. 7.

SourceData FS1 is the source file for Fig. S1.

SourceData FS3 is the source file for Fig. S3.

SourceData FS4 is the source file for Fig. S4.

SourceData FS5 is the source file for Fig. S5.

## Data Availability

All data are available in the main text or the supplemental materials. Further information and requests for resources and reagents should be directed to and will be fulfilled by the lead contact, Dimitri Krainc (dkrainc@nm.org).

## References

[bib1] Abu-Remaileh, M., G.A. Wyant, C. Kim, N.N. Laqtom, M. Abbasi, S.H. Chan, E. Freinkman, and D.M. Sabatini. 2017. Lysosomal metabolomics reveals V-ATPase- and mTOR-dependent regulation of amino acid efflux from lysosomes. Science. 358:807–813. 10.1126/science.aan6298 29074583 PMC5704967

[bib2] Ballabio, A., and J.S. Bonifacino. 2020. Lysosomes as dynamic regulators of cell and organismal homeostasis. Nat. Rev. Mol. Cell Biol. 21:101–118. 10.1038/s41580-019-0185-4 31768005

[bib3] Berndsen, K., P. Lis, W.M. Yeshaw, P.S. Wawro, R.S. Nirujogi, M. Wightman, T. Macartney, M. Dorward, A. Knebel, F. Tonelli, . 2019. PPM1H phosphatase counteracts LRRK2 signaling by selectively dephosphorylating Rab proteins. Elife. 8:e50416. 10.7554/eLife.50416 31663853 PMC6850886

[bib4] Bonet-Ponce, L., A. Beilina, C.D. Williamson, E. Lindberg, J.H. Kluss, S. Saez-Atienzar, N. Landeck, R. Kumaran, A. Mamais, C.K.E. Bleck, . 2020. LRRK2 mediates tubulation and vesicle sorting from lysosomes. Sci. Adv. 6:eabb2454. 10.1126/sciadv.abb2454 33177079 PMC7673727

[bib5] Britton, S., E. Dernoncourt, C. Delteil, C. Froment, O. Schiltz, B. Salles, P. Frit, and P. Calsou. 2014. DNA damage triggers SAF-A and RNA biogenesis factors exclusion from chromatin coupled to R-loops removal. Nucleic Acids Res. 42:9047–9062. 10.1093/nar/gku601 25030905 PMC4132723

[bib6] Burbulla, L.F., P. Song, J.R. Mazzulli, E. Zampese, Y.C. Wong, S. Jeon, D.P. Santos, J. Blanz, C.D. Obermaier, C. Strojny, . 2017. Dopamine oxidation mediates mitochondrial and lysosomal dysfunction in Parkinson’s disease. Science. 357:1255–1261. 10.1126/science.aam9080 28882997 PMC6021018

[bib7] Cai, S., Y. Wu, A. Guillén-Samander, W. Hancock-Cerutti, J. Liu, and P. De Camilli. 2022. In situ architecture of the lipid transport protein VPS13C at ER-lysosome membrane contacts. Proc. Natl. Acad. Sci. USA. 119:e2203769119. 10.1073/pnas.2203769119 35858323 PMC9303930

[bib8] Chen, E.I., D. McClatchy, S.K. Park, and J.R. Yates III. 2008. Comparisons of mass spectrometry compatible surfactants for global analysis of the mammalian brain proteome. Anal. Chem. 80:8694–8701. 10.1021/ac800606w 18937422 PMC2975600

[bib9] Chen, S., M.A. Roberts, C.Y. Chen, S. Markmiller, H.G. Wei, G.W. Yeo, J.G. Granneman, J.A. Olzmann, and S. Ferro-Novick. 2022. VPS13A and VPS13C influence lipid droplet abundance. Contact. 5:25152564221125613. 10.1177/25152564221125613 36147729 PMC9491623

[bib10] Chen, Y., Y. Deng, J. Zhang, L. Yang, X. Xie, and T. Xu. 2009. GDI-1 preferably interacts with Rab10 in insulin-stimulated GLUT4 translocation. Biochem. J. 422:229–235. 10.1042/BJ20090624 19570034 PMC2729519

[bib11] Cisneros, J., T.B. Belton, G.C. Shum, C.G. Molakal, and Y.C. Wong. 2022. Mitochondria-lysosome contact site dynamics and misregulation in neurodegenerative diseases. Trends Neurosci. 45:312–322. 10.1016/j.tins.2022.01.005 35249745 PMC8930467

[bib12] Cociorva, D., L.T. David, and J.R. Yates. 2007. Validation of tandem mass spectrometry database search results using DTASelect. Curr. Protoc. Bioinformatics. 13:13–14. 10.1002/0471250953.bi1304s16 18428785

[bib13] Darvish, H., P. Bravo, A. Tafakhori, L.J. Azcona, S. Ranji-Burachaloo, A.H. Johari, and C. Paisán-Ruiz. 2018. Identification of a large homozygous VPS13C deletion in a patient with early-onset Parkinsonism. Mov. Disord. 33:1968–1970. 10.1002/mds.27516 30452786 PMC6309582

[bib14] de Araujo, M.E.G., G. Liebscher, M.W. Hess, and L.A. Huber. 2020. Lysosomal size matters. Traffic. 21:60–75. 10.1111/tra.12714 31808235 PMC6972631

[bib15] Donkervoort, S., N. Krause, M. Dergai, P. Yun, J. Koliwer, S. Gorokhova, J. Geist Hauserman, B.B. Cummings, Y. Hu, R. Smith, . 2021. BET1 variants establish impaired vesicular transport as a cause for muscular dystrophy with epilepsy. EMBO Mol. Med. 13:e13787. 10.15252/emmm.202013787 34779586 PMC8649873

[bib16] Eguchi, T., T. Kuwahara, M. Sakurai, T. Komori, T. Fujimoto, G. Ito, S.I. Yoshimura, A. Harada, M. Fukuda, M. Koike, and T. Iwatsubo. 2018. LRRK2 and its substrate Rab GTPases are sequentially targeted onto stressed lysosomes and maintain their homeostasis. Proc. Natl. Acad. Sci. USA. 115:E9115–E9124. 10.1073/pnas.1812196115 30209220 PMC6166828

[bib17] Eng, J.K., A.L. McCormack, and J.R. Yates. 1994. An approach to correlate tandem mass spectral data of peptides with amino acid sequences in a protein database. J. Am. Soc. Mass Spectrom. 5:976–989. 10.1016/1044-0305(94)80016-2 24226387

[bib18] Falcón-Pérez, J.M., R. Nazarian, C. Sabatti, and E.C. Dell’Angelica. 2005. Distribution and dynamics of Lamp1-containing endocytic organelles in fibroblasts deficient in BLOC-3. J. Cell Sci. 118:5243–5255. 10.1242/jcs.02633 16249233

[bib19] Friedman, J.R., L.L. Lackner, M. West, J.R. DiBenedetto, J. Nunnari, and G.K. Voeltz. 2011. ER tubules mark sites of mitochondrial division. Science. 334:358–362. 10.1126/science.1207385 21885730 PMC3366560

[bib20] Gillingham, A.K., J. Bertram, F. Begum, and S. Munro. 2019. In vivo identification of GTPase interactors by mitochondrial relocalization and proximity biotinylation. Elife. 8:e45916. 10.7554/eLife.45916 31294692 PMC6639074

[bib21] Gu, X., C. Li, Y. Chen, R. Ou, B. Cao, Q. Wei, Y. Hou, L. Zhang, W. Song, B. Zhao, . 2020. Mutation screening and burden analysis of VPS13C in Chinese patients with early-onset Parkinson’s disease. Neurobiol. Aging. 94:311 e311–311 e314. 10.1016/j.neurobiolaging.2020.05.005 32507414

[bib22] Hancock-Cerutti, W., Z. Wu, P. Xu, N. Yadavalli, M. Leonzino, A.K. Tharkeshwar, S.M. Ferguson, G.S. Shadel, and P. De Camilli. 2022. ER-lysosome lipid transfer protein VPS13C/PARK23 prevents aberrant mtDNA-dependent STING signaling. J. Cell Biol. 221:e202106046. 10.1083/jcb.202106046 35657605 PMC9170524

[bib23] He, L., J. Diedrich, Y.Y. Chu, and J.R. Yates III. 2015. Extracting accurate precursor information for tandem mass spectra by RawConverter. Anal. Chem. 87:11361–11367. 10.1021/acs.analchem.5b02721 26499134 PMC4777630

[bib24] Herbst, S., P. Campbell, J. Harvey, E.M. Bernard, V. Papayannopoulos, N.W. Wood, H.R. Morris, and M.G. Gutierrez. 2020. LRRK2 activation controls the repair of damaged endomembranes in macrophages. EMBO J. 39:e104494. 10.15252/embj.2020104494 32643832 PMC7507578

[bib25] Homma, Y., S. Hiragi, and M. Fukuda. 2021. Rab family of small GTPases: An updated view on their regulation and functions. FEBS J. 288:36–55. 10.1111/febs.15453 32542850 PMC7818423

[bib26] Hook, S.C., A. Chadt, K.J. Heesom, S. Kishida, H. Al-Hasani, J.M. Tavaré, and E.C. Thomas. 2020. TBC1D1 interacting proteins, VPS13A and VPS13C, regulate GLUT4 homeostasis in C2C12 myotubes. Sci. Rep. 10:17953. 10.1038/s41598-020-74661-1 33087848 PMC7578007

[bib27] Hopfner, F., S.H. Mueller, S. Szymczak, O. Junge, L. Tittmann, S. May, K. Lohmann, H. Grallert, W. Lieb, K. Strauch, . 2020. Rare variants in specific lysosomal genes are associated with Parkinson’s disease. Mov. Disord. 35:1245–1248. 10.1002/mds.28037 32267580

[bib28] Huttlin, E.L., R.J. Bruckner, J.A. Paulo, J.R. Cannon, L. Ting, K. Baltier, G. Colby, F. Gebreab, M.P. Gygi, H. Parzen, . 2017. Architecture of the human interactome defines protein communities and disease networks. Nature. 545:505–509. 10.1038/nature22366 28514442 PMC5531611

[bib29] Ito, Y., S. Nakamura, N. Sugimoto, T. Shigemori, Y. Kato, M. Ohno, S. Sakuma, K. Ito, H. Kumon, H. Hirose, . 2018. Turbulence activates platelet biogenesis to enable clinical scale Ex Vivo production. Cell. 174:636–648.e18. 10.1016/j.cell.2018.06.011 30017246

[bib30] Jansen, I.E., H. Ye, S. Heetveld, M.C. Lechler, H. Michels, R.I. Seinstra, S.J. Lubbe, V. Drouet, S. Lesage, E. Majounie, . 2017. Discovery and functional prioritization of Parkinson’s disease candidate genes from large-scale whole exome sequencing. Genome Biol. 18:22. 10.1186/s13059-017-1147-9 28137300 PMC5282828

[bib31] Jiang, X., C. Zhang, J. Chen, S. Choi, Y. Zhou, M. Zhao, X. Song, X. Chen, M. Maletić-Savatić, T. Palzkill, . 2019. Quantitative real-time imaging of glutathione with subcellular resolution. Antioxid. Redox Signal. 30:1900–1910. 10.1089/ars.2018.7605 30358421 PMC6486671

[bib32] Kim, S., R. Coukos, F. Gao, and D. Krainc. 2022. Dysregulation of organelle membrane contact sites in neurological diseases. Neuron. 110:2386–2408. 10.1016/j.neuron.2022.04.020 35561676 PMC9357093

[bib33] Kim, S., Y.C. Wong, F. Gao, and D. Krainc. 2021. Dysregulation of mitochondria-lysosome contacts by GBA1 dysfunction in dopaminergic neuronal models of Parkinson’s disease. Nat. Commun. 12:1807. 10.1038/s41467-021-22113-3 33753743 PMC7985376

[bib34] Kluss, J.H., A. Beilina, C.D. Williamson, P.A. Lewis, M.R. Cookson, and L. Bonet-Ponce. 2022. Lysosomal positioning regulates Rab10 phosphorylation at LRRK2^+^ lysosomes. Proc. Natl. Acad. Sci. USA. 119:e2205492119. 10.1073/pnas.2205492119 36256825 PMC9618077

[bib35] Kriks, S., J.-W. Shim, J. Piao, Y.M. Ganat, D.R. Wakeman, Z. Xie, L. Carrillo-Reid, G. Auyeung, C. Antonacci, A. Buch, . 2011. Dopamine neurons derived from human ES cells efficiently engraft in animal models of Parkinson’s disease. Nature. 480:547–551. 10.1038/nature10648 22056989 PMC3245796

[bib36] Kumar, N., M. Leonzino, W. Hancock-Cerutti, F.A. Horenkamp, P. Li, J.A. Lees, H. Wheeler, K.M. Reinisch, and P. De Camilli. 2018. VPS13A and VPS13C are lipid transport proteins differentially localized at ER contact sites. J. Cell Biol. 217:3625–3639. 10.1083/jcb.201807019 30093493 PMC6168267

[bib37] Kuwahara, T., K. Funakawa, T. Komori, M. Sakurai, G. Yoshii, T. Eguchi, M. Fukuda, and T. Iwatsubo. 2020. Roles of lysosomotropic agents on LRRK2 activation and Rab10 phosphorylation. Neurobiol. Dis. 145:105081. 10.1016/j.nbd.2020.105081 32919031

[bib38] Lesage, S., V. Drouet, E. Majounie, V. Deramecourt, M. Jacoupy, A. Nicolas, F. Cormier-Dequaire, S.M. Hassoun, C. Pujol, S. Ciura, . 2016. Loss of VPS13C function in autosomal-recessive parkinsonism causes mitochondrial dysfunction and increases PINK1/parkin-dependent mitophagy. Am. J. Hum. Genet. 98:500–513. 10.1016/j.ajhg.2016.01.014 26942284 PMC4800038

[bib39] Lie, P.P.Y., and R.A. Nixon. 2019. Lysosome trafficking and signaling in health and neurodegenerative diseases. Neurobiol. Dis. 122:94–105. 10.1016/j.nbd.2018.05.015 29859318 PMC6381838

[bib40] Nalls, M.A., C. Blauwendraat, C.L. Vallerga, K. Heilbron, S. Bandres-Ciga, D. Chang, M. Tan, D.A. Kia, A.J. Noyce, A. Xue, . 2019. Identification of novel risk loci, causal insights, and heritable risk for Parkinson’s disease: A meta-analysis of genome-wide association studies. Lancet Neurol. 18:1091–1102. 10.1016/S1474-4422(19)30320-5 31701892 PMC8422160

[bib41] Nalls, M.A., N. Pankratz, C.M. Lill, C.B. Do, D.G. Hernandez, M. Saad, A.L. DeStefano, E. Kara, J. Bras, M. Sharma, . 2014. Large-scale meta-analysis of genome-wide association data identifies six new risk loci for Parkinson’s disease. Nat. Genet. 46:989–993. 10.1038/ng.3043 25064009 PMC4146673

[bib42] Navarro-Romero, A., M. Montpeyó, and M. Martinez-Vicente. 2020. The emerging role of the lysosome in Parkinson’s disease. Cells. 9:2399. 10.3390/cells9112399 33147750 PMC7692401

[bib43] Nguyen, M., and D. Krainc. 2018. LRRK2 phosphorylation of auxilin mediates synaptic defects in dopaminergic neurons from patients with Parkinson’s disease. Proc. Natl. Acad. Sci. USA. 115:5576–5581. 10.1073/pnas.1717590115 29735704 PMC6003526

[bib44] Nguyen, M., Y.C. Wong, D. Ysselstein, A. Severino, and D. Krainc. 2019. Synaptic, mitochondrial, and lysosomal dysfunction in Parkinson’s disease. Trends Neurosci. 42:140–149. 10.1016/j.tins.2018.11.001 30509690 PMC6452863

[bib45] Pan, H., Z. Liu, J. Ma, Y. Li, Y. Zhao, X. Zhou, Y. Xiang, Y. Wang, X. Zhou, R. He, . 2023. Genome-wide association study using whole-genome sequencing identifies risk loci for Parkinson’s disease in Chinese population. NPJ Parkinsons Dis. 9:22. 10.1038/s41531-023-00456-6 36759515 PMC9911746

[bib46] Peng, J., J.E. Elias, C.C. Thoreen, L.J. Licklider, and S.P. Gygi. 2003. Evaluation of multidimensional chromatography coupled with tandem mass spectrometry (LC/LC-MS/MS) for large-scale protein analysis: The yeast proteome. J. Proteome Res. 2:43–50. 10.1021/pr025556v 12643542

[bib47] Peng, W., L.F. Schröder, P. Song, Y.C. Wong, and D. Krainc. 2023. Parkin regulates amino acid homeostasis at mitochondria-lysosome (M/L) contact sites in Parkinson’s disease. Sci. Adv. 9:eadh3347. 10.1126/sciadv.adh3347 37467322 PMC10355824

[bib48] Poewe, W., K. Seppi, C.M. Tanner, G.M. Halliday, P. Brundin, J. Volkmann, A.-E. Schrag, and A.E. Lang. 2017. Parkinson disease. Nat. Rev. Dis. Primers. 3:17013. 10.1038/nrdp.2017.13 28332488

[bib49] Quitterer, U., X. Fu, A. Pohl, K.M. Bayoumy, A. Langer, and S. AbdAlla. 2019. Beta-Arrestin1 prevents preeclampsia by downregulation of mechanosensitive AT1-B2 receptor heteromers. Cell. 176:318–333.e19. 10.1016/j.cell.2018.10.050 30503206

[bib50] Radulovic, M., and H. Stenmark. 2020. LRRK2 to the rescue of damaged endomembranes. EMBO J. 39:e106162. 10.15252/embj.2020106162 32803793 PMC7507336

[bib51] Roney, J.C., X.T. Cheng, and Z.H. Sheng. 2022. Neuronal endolysosomal transport and lysosomal functionality in maintaining axonostasis. J. Cell Biol. 221:221. 10.1083/jcb.202111077 PMC8932522 35142819

[bib52] Rzomp, K.A., L.D. Scholtes, B.J. Briggs, G.R. Whittaker, and M.A. Scidmore. 2003. Rab GTPases are recruited to chlamydial inclusions in both a species-dependent and species-independent manner. Infect. Immun. 71:5855–5870. 10.1128/IAI.71.10.5855-5870.2003 14500507 PMC201052

[bib53] Schapansky, J., S. Khasnavis, M.P. DeAndrade, J.D. Nardozzi, S.R. Falkson, J.D. Boyd, J.B. Sanderson, T. Bartels, H.L. Melrose, and M.J. LaVoie. 2018. Familial knockin mutation of LRRK2 causes lysosomal dysfunction and accumulation of endogenous insoluble α-synuclein in neurons. Neurobiol. Dis. 111:26–35. 10.1016/j.nbd.2017.12.005 29246723 PMC5803451

[bib54] Schormair, B., D. Kemlink, B. Mollenhauer, O. Fiala, G. Machetanz, J. Roth, R. Berutti, T.M. Strom, B. Haslinger, C. Trenkwalder, . 2018. Diagnostic exome sequencing in early-onset Parkinson’s disease confirms VPS13C as a rare cause of autosomal-recessive Parkinson’s disease. Clin. Genet. 93:603–612. 10.1111/cge.13124 28862745

[bib55] Sherer, N.M., M.J. Lehmann, L.F. Jimenez-Soto, A. Ingmundson, S.M. Horner, G. Cicchetti, P.G. Allen, M. Pypaert, J.M. Cunningham, and W. Mothes. 2003. Visualization of retroviral replication in living cells reveals budding into multivesicular bodies. Traffic. 4:785–801. 10.1034/j.1600-0854.2003.00135.x 14617360

[bib56] Smolders, S., S. Philtjens, D. Crosiers, A. Sieben, E. Hens, B. Heeman, S. Van Mossevelde, P. Pals, B. Asselbergh, R. Dos Santos Dias, . 2021. Contribution of rare homozygous and compound heterozygous VPS13C missense mutations to dementia with Lewy bodies and Parkinson’s disease. Acta Neuropathol. Commun. 9:25. 10.1186/s40478-021-01121-w 33579389 PMC7881566

[bib57] Song, P., W. Peng, V. Sauve, R. Fakih, Z. Xie, D. Ysselstein, T. Krainc, Y.C. Wong, N.E. Mencacci, J.N. Savas, . 2023. Parkinson’s disease-linked parkin mutation disrupts recycling of synaptic vesicles in human dopaminergic neurons. Neuron. 111:3775–3788.e7. 10.1016/j.neuron.2023.08.018 37716354 PMC11977536

[bib58] Stefely, J.A., Y. Zhang, E.C. Freiberger, N.W. Kwiecien, H.E. Thomas, A.M. Davis, N.D. Lowry, C.E. Vincent, E. Shishkova, N.A. Clark, . 2020. Mass spectrometry proteomics reveals a function for mammalian CALCOCO1 in MTOR-regulated selective autophagy. Autophagy. 16:2219–2237. 10.1080/15548627.2020.1719746 31971854 PMC7751563

[bib59] Steger, M., F. Tonelli, G. Ito, P. Davies, M. Trost, M. Vetter, S. Wachter, E. Lorentzen, G. Duddy, S. Wilson, . 2016. Phosphoproteomics reveals that Parkinson’s disease kinase LRRK2 regulates a subset of Rab GTPases. Elife. 5:e12813. 10.7554/eLife.12813 26824392 PMC4769169

[bib60] Tabb, D.L., W.H. McDonald, and J.R. Yates III. 2002. DTASelect and contrast: Tools for assembling and comparing protein identifications from shotgun proteomics. J. Proteome Res. 1:21–26. 10.1021/pr015504q 12643522 PMC2811961

[bib61] Takahashi, K., K. Tanabe, M. Ohnuki, M. Narita, T. Ichisaka, K. Tomoda, and S. Yamanaka. 2007. Induction of pluripotent stem cells from adult human fibroblasts by defined factors. Cell. 131:861–872. 10.1016/j.cell.2007.11.019 18035408

[bib62] Wallings, R.L., S.W. Humble, M.E. Ward, and R. Wade-Martins. 2019. Lysosomal dysfunction at the centre of Parkinson’s disease and frontotemporal dementia/amyotrophic lateral sclerosis. Trends Neurosci. 42:899–912. 10.1016/j.tins.2019.10.002 31704179 PMC6931156

[bib63] Wilson, E.L., and E. Metzakopian. 2021. ER-Mitochondria contact sites in neurodegeneration: Genetic screening approaches to investigate novel disease mechanisms. Cell Death Differ. 28:1804–1821. 10.1038/s41418-020-00705-8 33335290 PMC8185109

[bib64] Wong, Y.C., S. Kim, J. Cisneros, C.G. Molakal, P. Song, S.J. Lubbe, and D. Krainc. 2022. Mid51/Fis1 mitochondrial oligomerization complex drives lysosomal untethering and network dynamics. J. Cell Biol. 221:e202206140. 10.1083/jcb.202206140 36044022 PMC9437119

[bib65] Wong, Y.C., D. Ysselstein, and D. Krainc. 2018. Mitochondria-lysosome contacts regulate mitochondrial fission via RAB7 GTP hydrolysis. Nature. 554:382–386. 10.1038/nature25486 29364868 PMC6209448

[bib66] Xu, T., J.D. Venable, S.K. Park, D. Cociorva, B. Lu, L. Liao, J. Wohlschlegel, J. Hewel, and J. Yates. 2006. ProLuCID, a fast and sensitive tandem mass spectra-based protein identification program. Mol. Cell. Proteomics. 5:S174.

[bib67] Yang, R.Y., H. Xue, L. Yu, A. Velayos-Baeza, A.P. Monaco, and F.T. Liu. 2016. Identification of VPS13C as a galectin-12-binding protein that regulates galectin-12 protein stability and adipogenesis. PLoS One. 11:e0153534. 10.1371/journal.pone.0153534 27073999 PMC4830523

[bib68] Zerial, M., and H. McBride. 2001. Rab proteins as membrane organizers. Nat. Rev. Mol. Cell Biol. 2:107–117. 10.1038/35052055 11252952

