# Supplementary figures and images for: VPS13C regulates phospho-Rab10-mediated lysosomal function in human dopaminergic neurons
Source: J Cell Biol. 2024 Feb 15;223(5):e202304042. doi: 10.1083/jcb.202304042 (PMC10868123; doi:10.1083/jcb.202304042)

Figure 1

1A

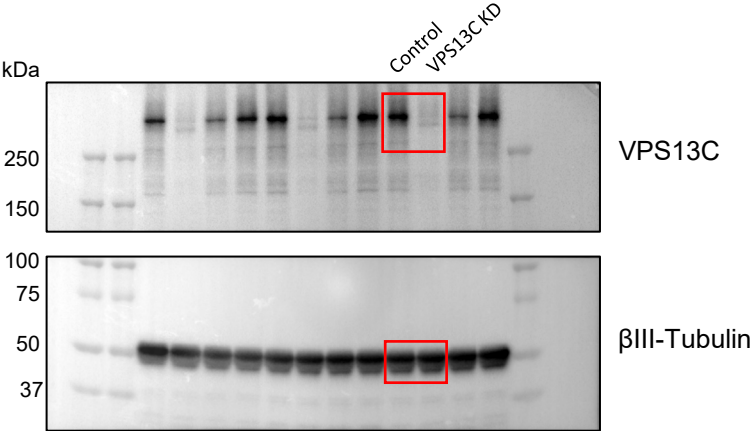

Supplement: SourceData F1 — is the source file for Fig. 1. [file JCB_202304042_SourceDataF1.pdf]

Figure 4

4A

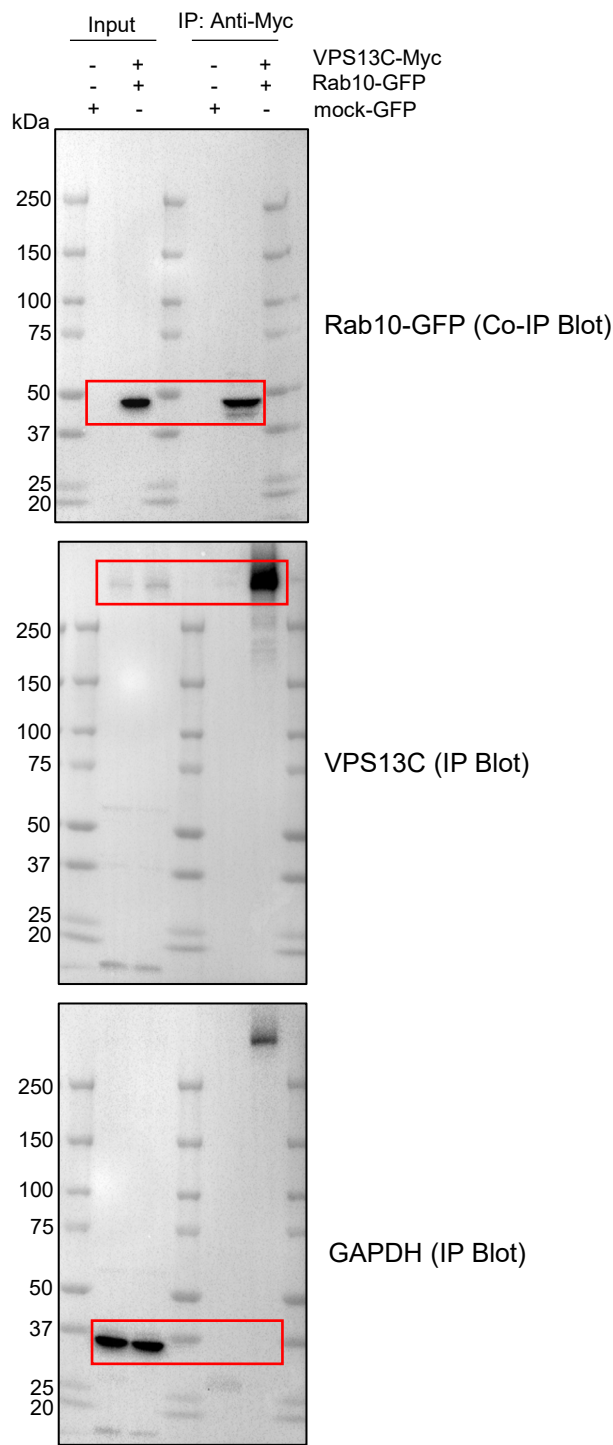

Figure 4

4B

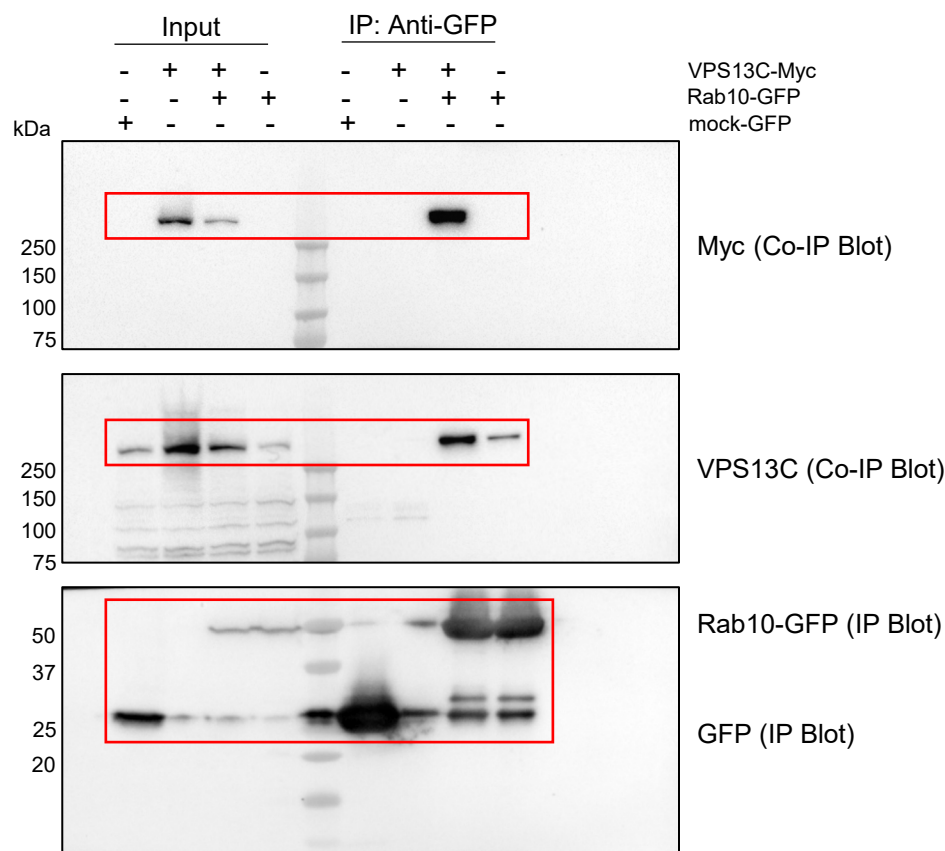

Figure 4

4D

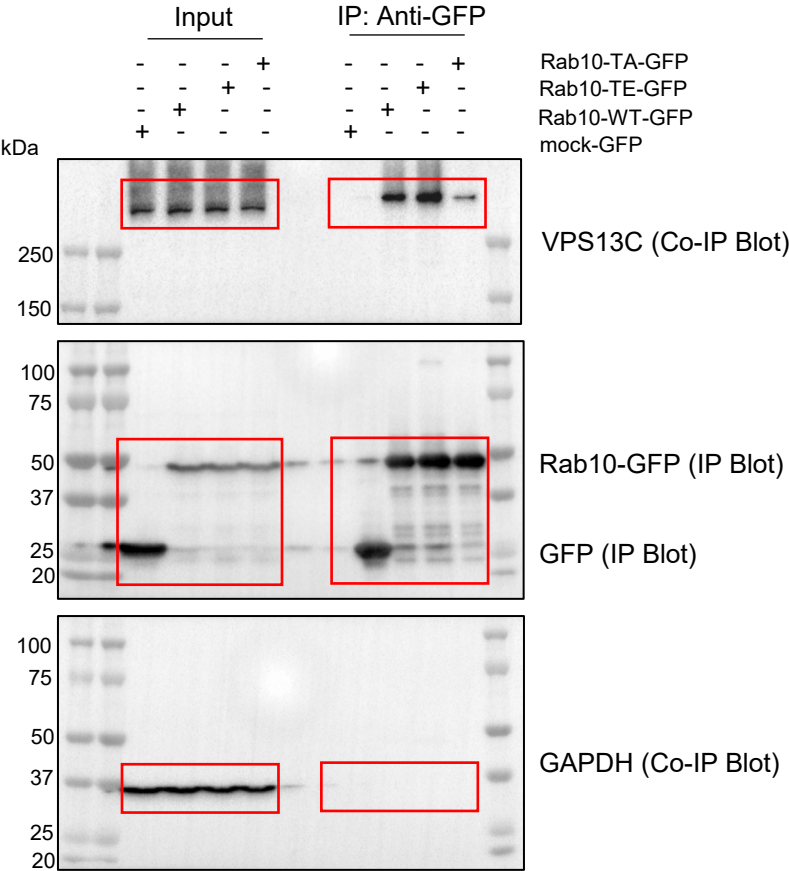

Supplement: SourceData F4 — is the source file for Fig. 4. [file JCB_202304042_SourceDataF4.pdf]

Figure 5

5G

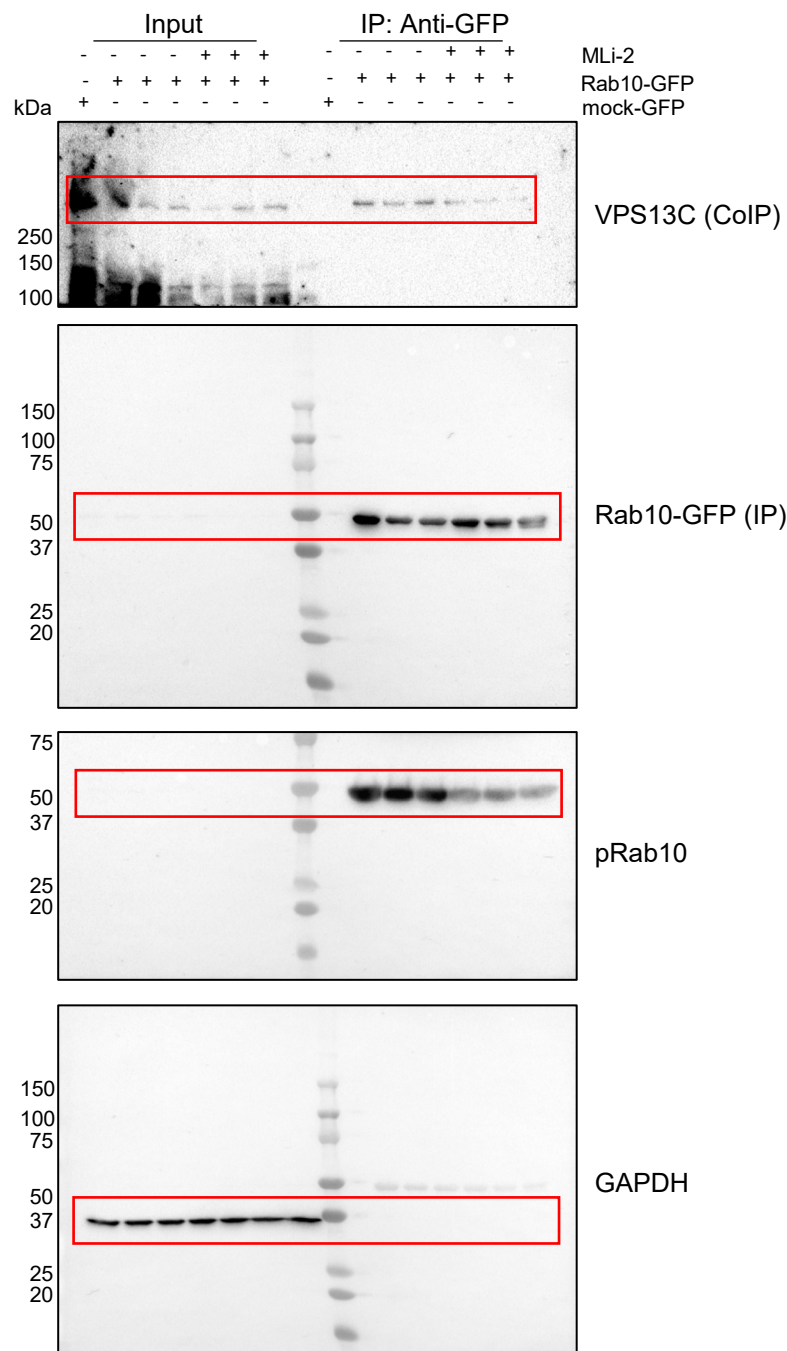

Supplement: SourceData F5 — is the source file for Fig. 5. [file JCB_202304042_SourceDataF5.pdf]

Figure 6

6A

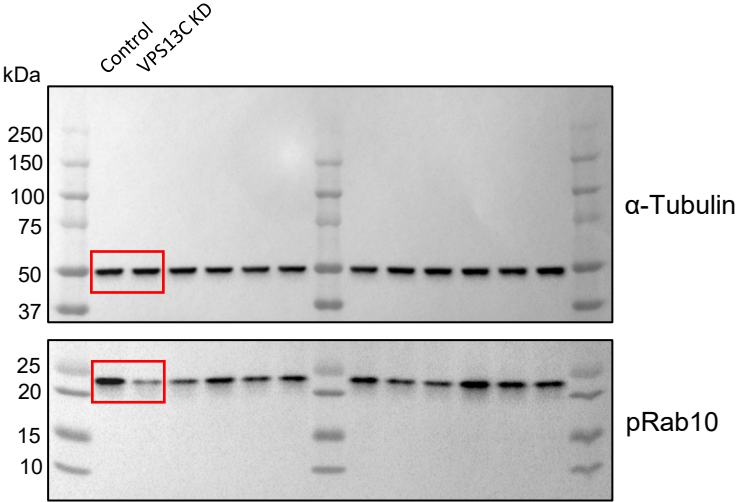

6B

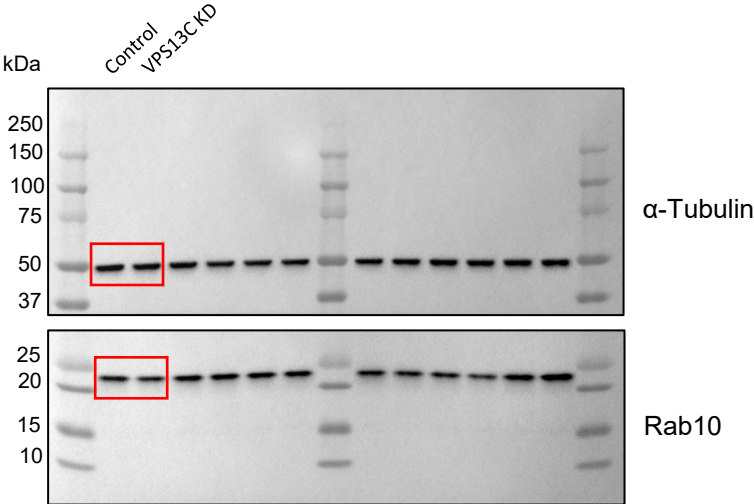

Figure 6

6E

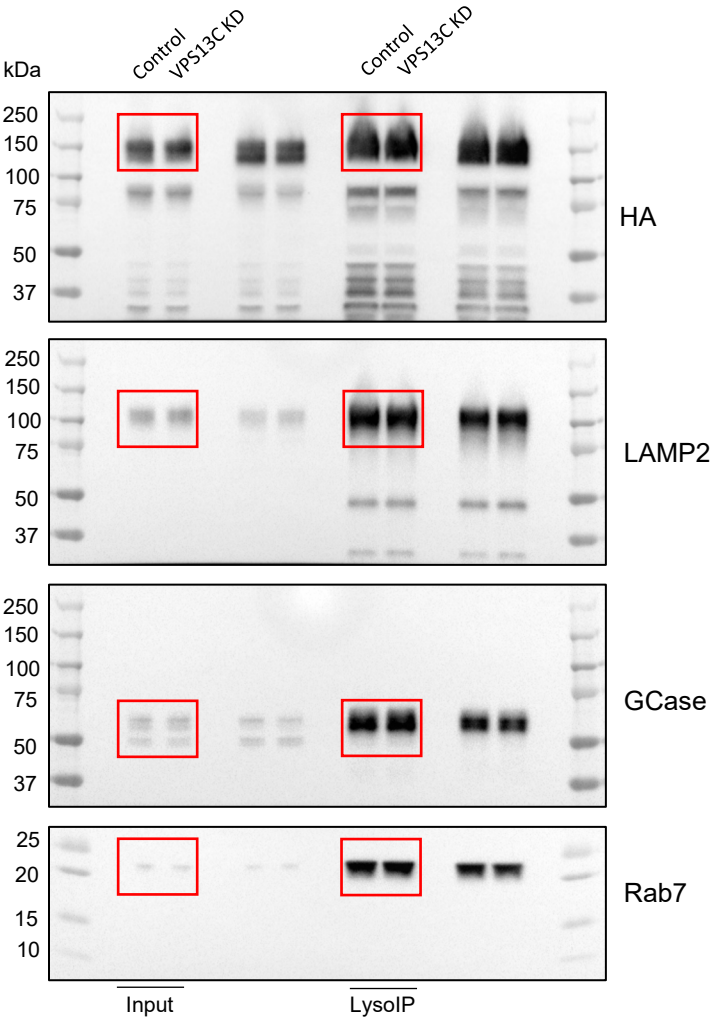

Figure 6

6F

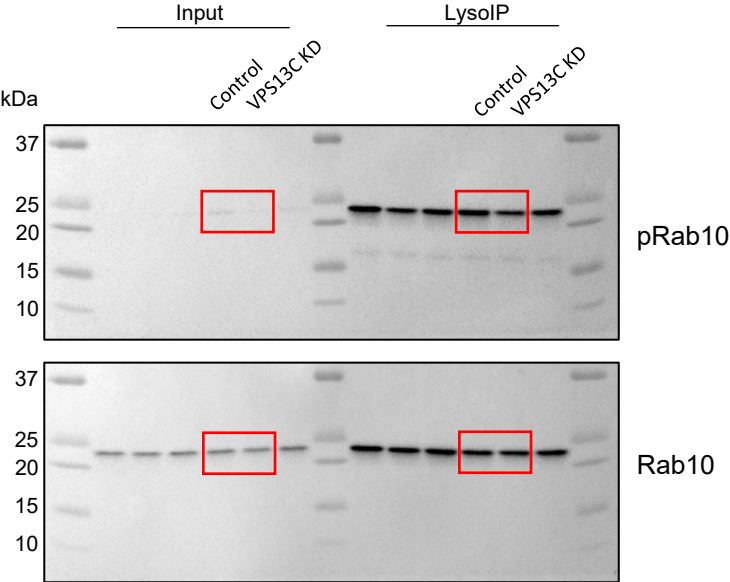

Figure 6

6H

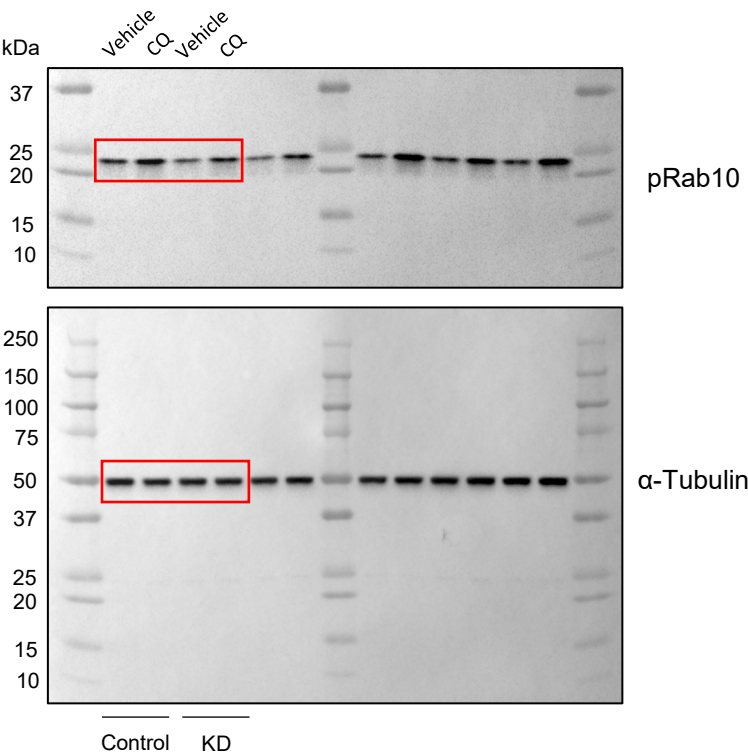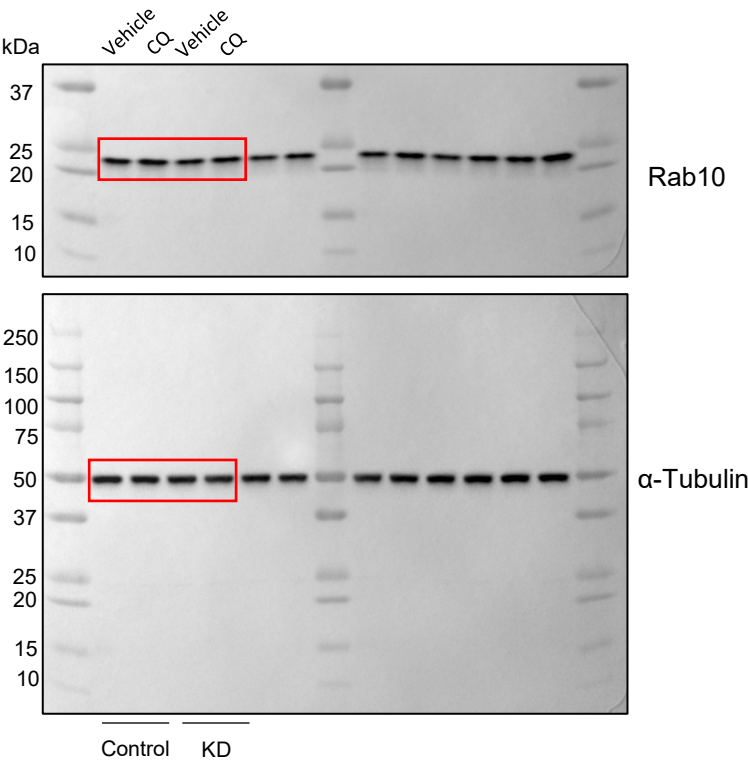

Figure 6

6J

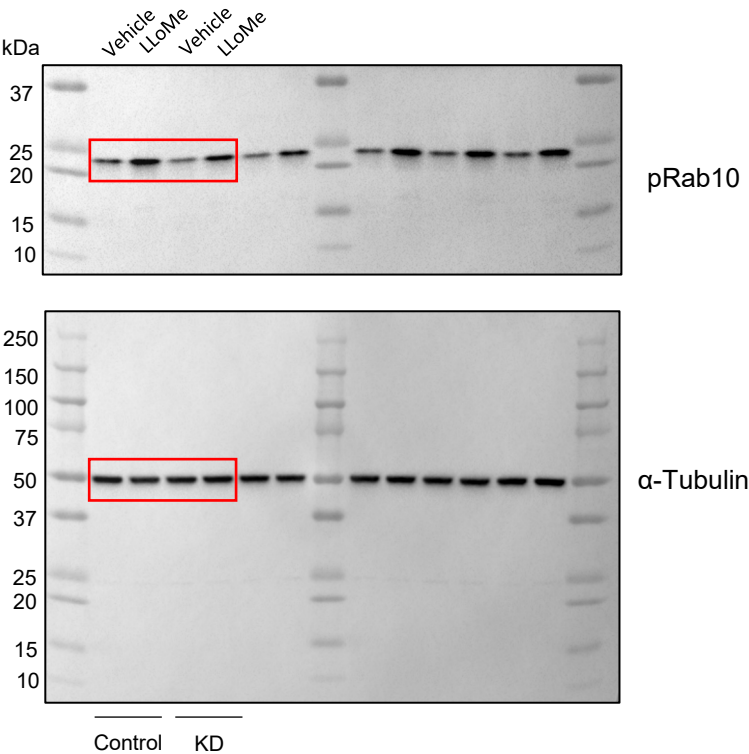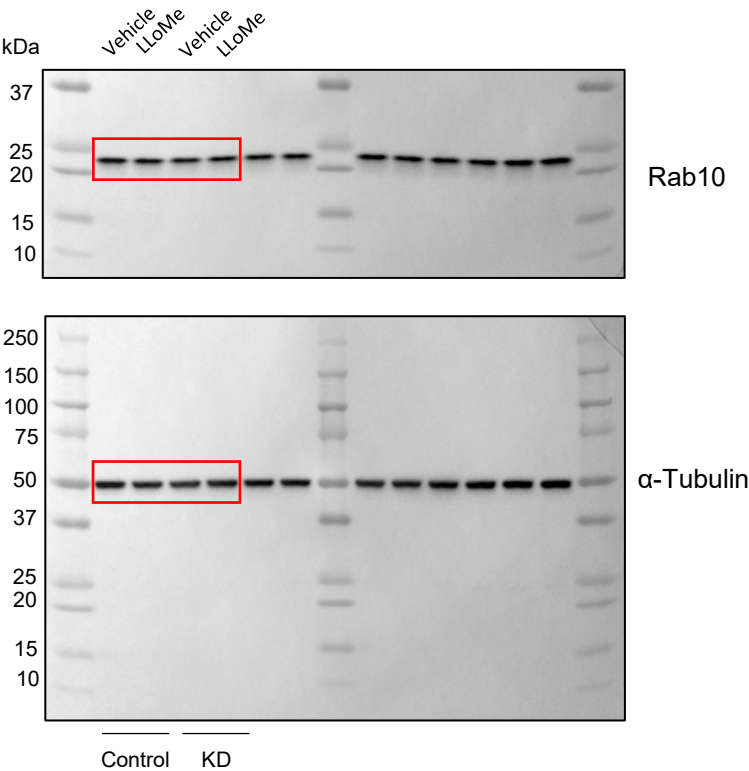

Supplement: SourceData F6 — is the source file for Fig. 6. [file JCB_202304042_SourceDataF6.pdf]

Figure 7

7A

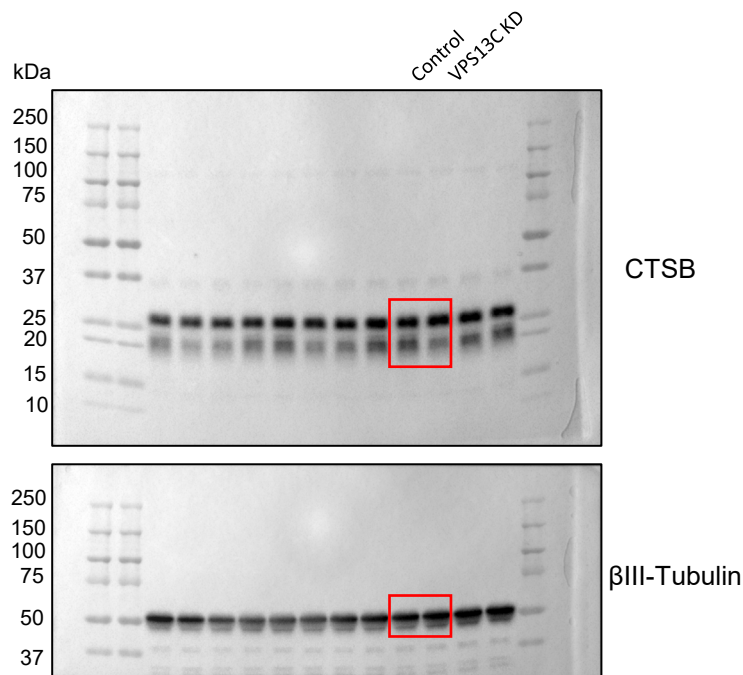

7C

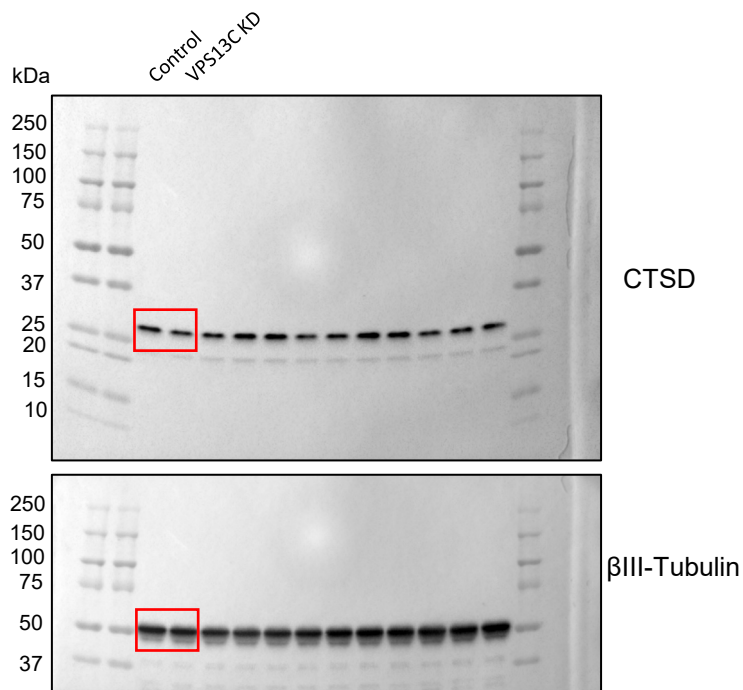

Supplement: SourceData F7 — is the source file for Fig. 7. [file JCB_202304042_SourceDataF7.pdf]

Supplement Figure 1

S1E

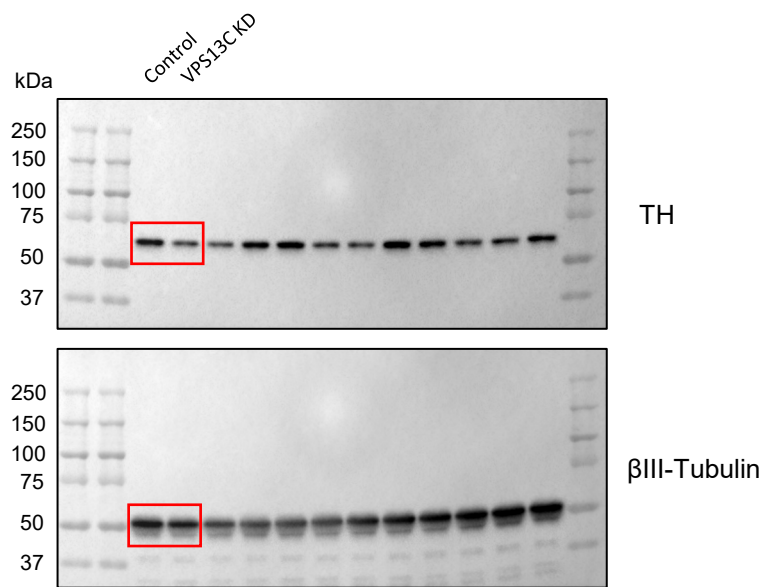

S1G

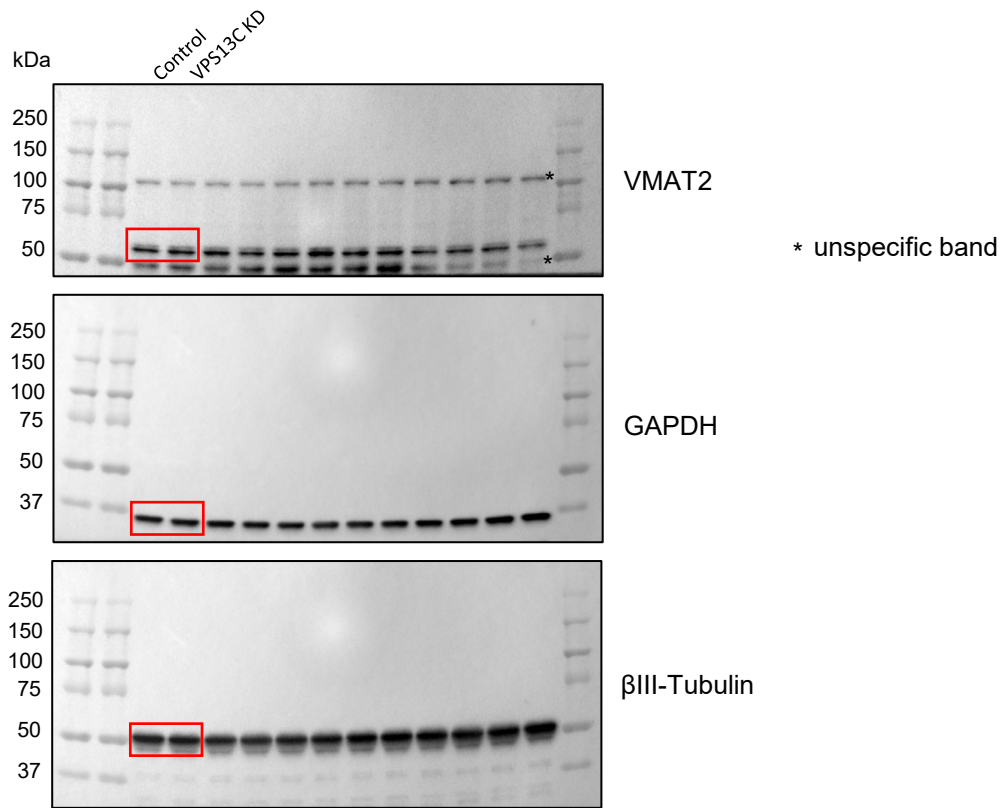

Supplement: SourceData FS1 — is the source file for Fig. S1. [file JCB_202304042_SourceDataFS1.pdf]

Supplement Figure 3

S3E

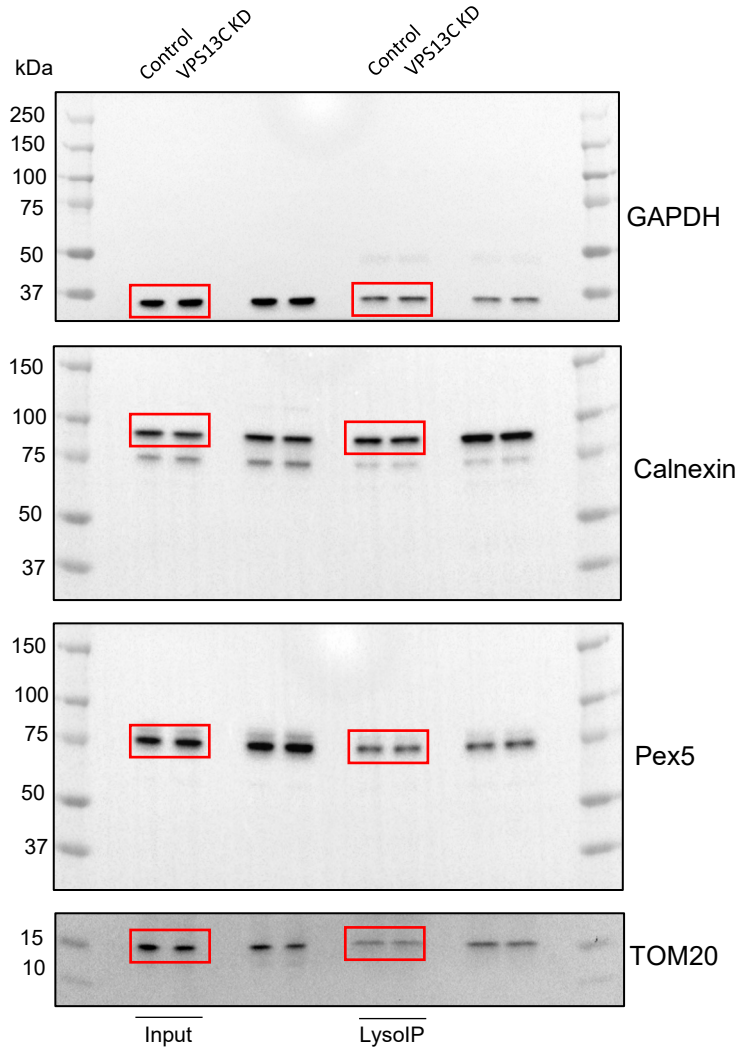

Supplement Figure 3

S3I

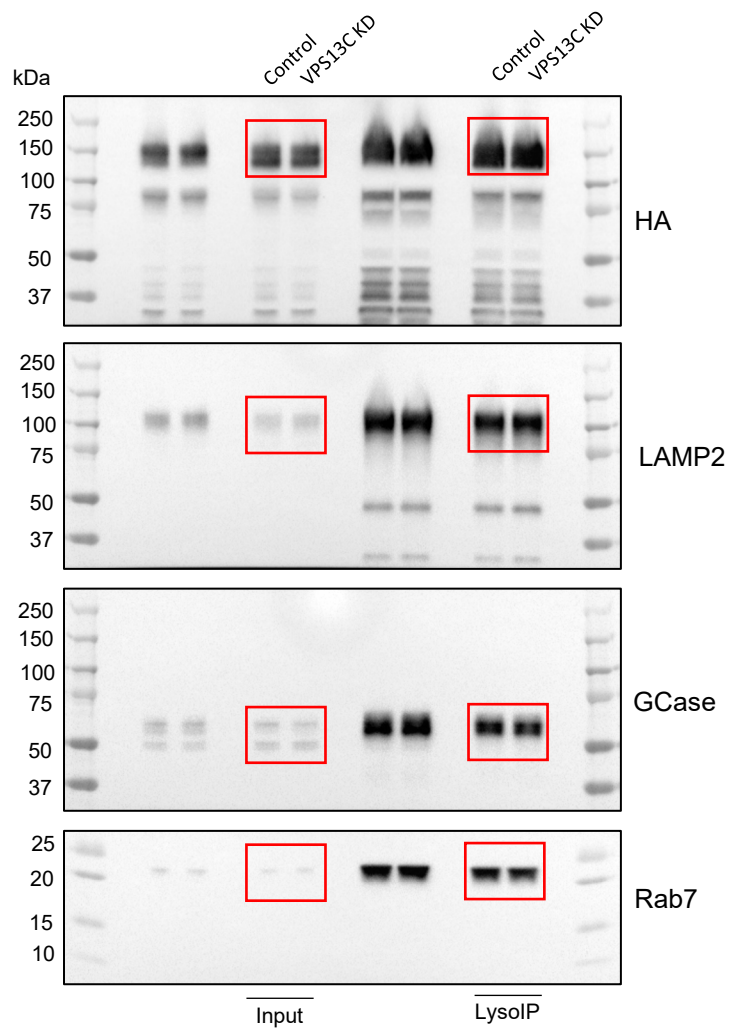

Supplement Figure 3

S3J

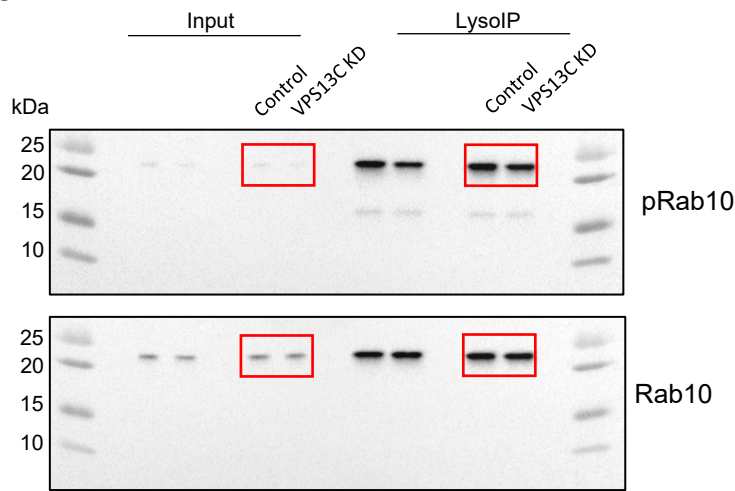

Supplement: SourceData FS3 — is the source file for Fig. S3. [file JCB_202304042_SourceDataFS3.pdf]

Supplement Figure 4

S4A

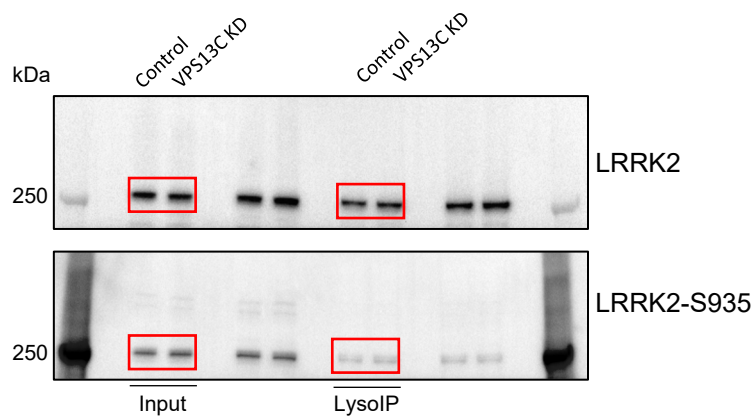

S4D

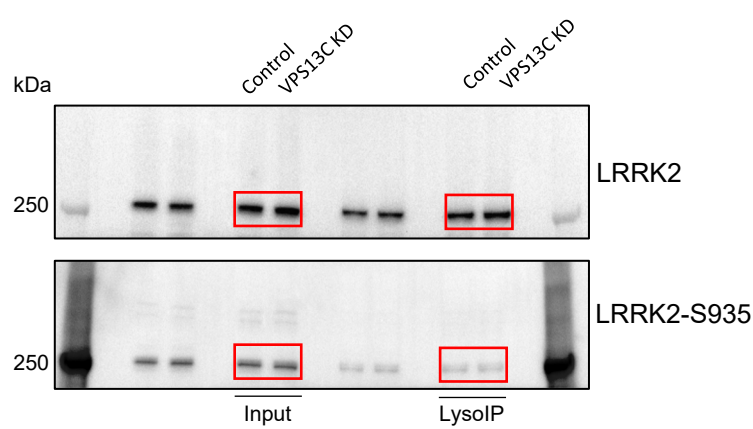

Supplement Figure 4

S4G

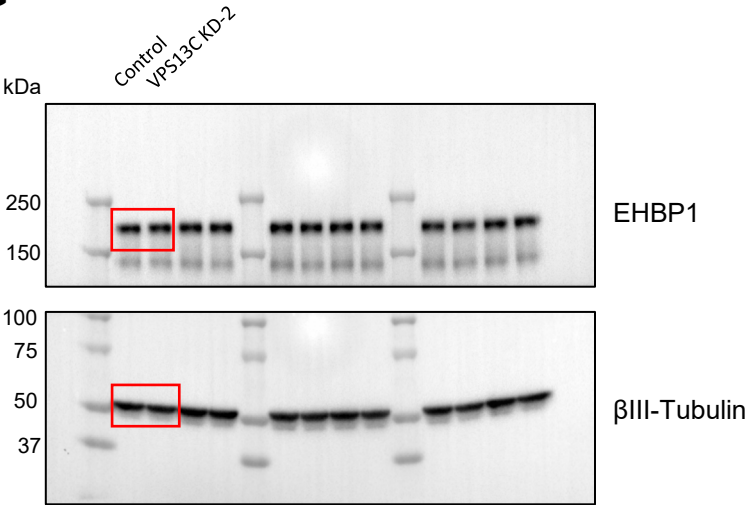

S4I

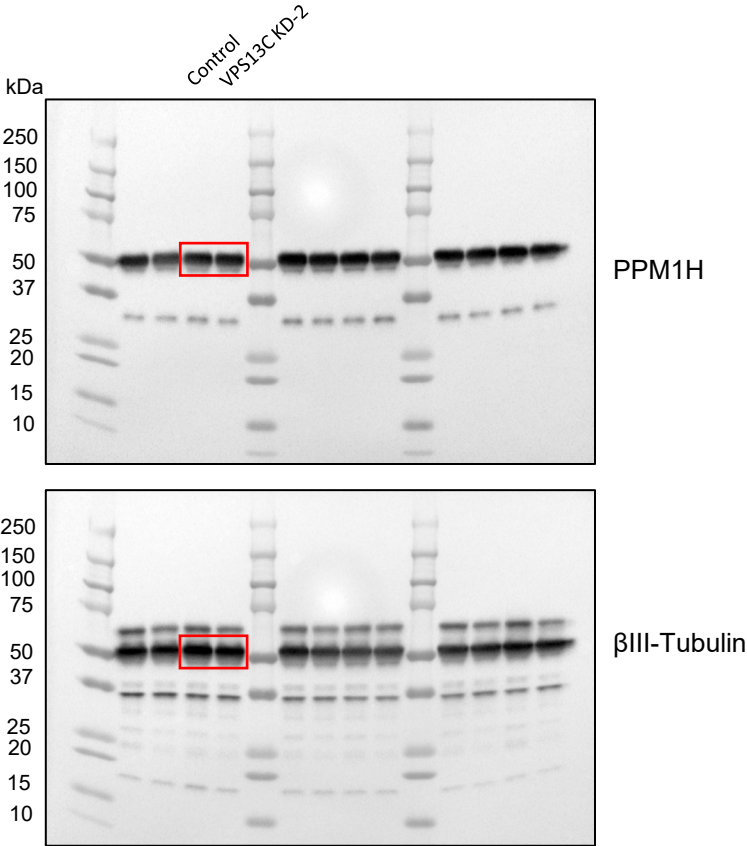

Supplement: SourceData FS4 — is the source file for Fig. S4. [file JCB_202304042_SourceDataFS4.pdf]

# Supplement Figure 5

## S5A

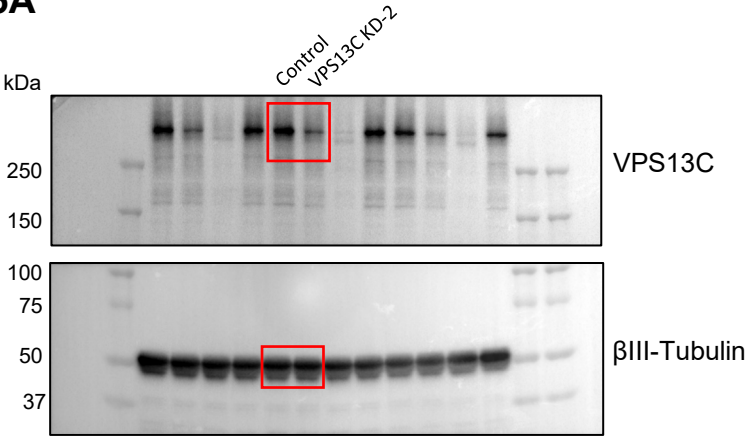

## S5Q

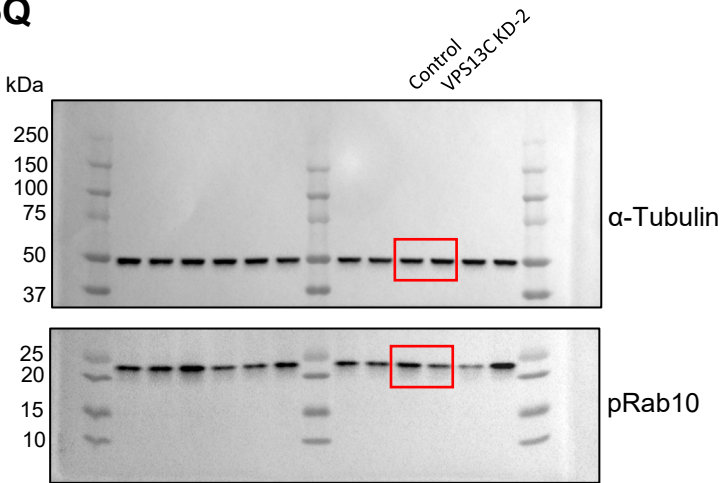

## S5R

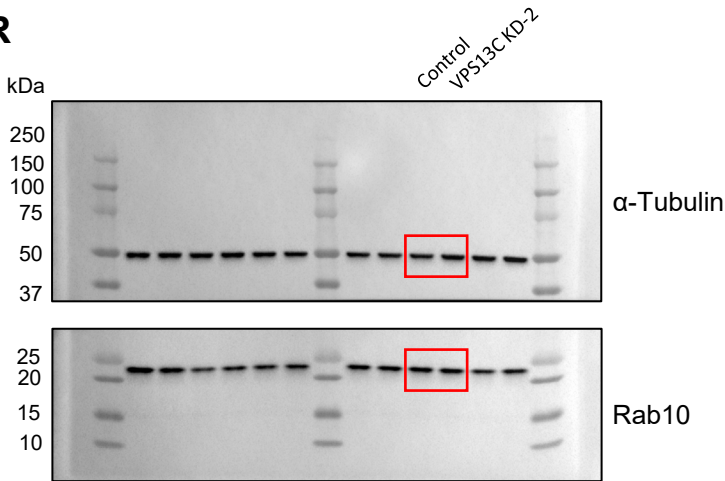

Supplement: SourceData FS5 — is the source file for Fig. S5. [file JCB_202304042_SourceDataFS5.pdf]
